# A rapid assessment methodology for quantifying and visualizing functional landscape connectivity

**DOI:** 10.3389/fcosc.2024.1412888

**Published:** 2024-07-04

**Authors:** Nathan H. Schumaker

**Affiliations:** Pacific Ecological Systems Division, US Environmental Protection Agency, Corvallis, OR, United States

**Keywords:** connectivity, movement, simulation model, circuit theory, graph theory, dispersal kernel

## Abstract

**Context:**

The number of publications that evaluate or use landscape connectivity has grown dramatically in recent years. But the biological realism of common connectivity assessments remains limited. To address this shortcoming, I introduce a flexible methodology for evaluating functional landscape connectivity that can be quick to implement, biologically nuanced, and straightforward to interpret.

**Methods:**

I combined a US Fish and Wildlife Service land cover map with information from existing empirical studies to develop a movement simulator for the Fender’s blue butterfly, an endangered species in Oregon, USA. I use the resulting butterfly model to explore the concepts and mechanics behind my novel connectivity assessment methodology.

**Results:**

My methods are able to identify clusters of connected resource patches, quantify and visualize movement rates between patches, and identify opportunities for enhancing connectivity through restoration and mitigation. My results include an emergent dispersal kernel that captures the influence of movement behavior on connectivity.

**Discussion:**

The methods I introduce are capable of generating detailed yet practical connectivity analyses that can incorporate considerable biological and behavioral realism. My approach is simple to implement, and the requisite data can be modest. The toolkit I developed has the potential to standardize connectivity assessments that use either real or simulated movement data.

## Introduction

Connectivity assessments are assertions about a landscape’s ability to facilitate or impede movement (Taylor et al., 2006). In this context, the things doing the moving are typically living organisms, but could also include viral pathogens, inert objects, flows of energy, ideas, and so on. And any measure of connectivity will be context-specific, as the same landscape can be highly connected for one species or quantity, while being poorly connected for others. Connectivity can be a well-defined and objectively interpretable attribute of fractal-dimensioned networks (Schmadel et al., 2018; Sarker et al., 2019; Xingyuan et al., 2023; Clauzel et al., 2024), but tends to be difficult to assess in two or three dimensional landscapes (Guarenghi et al., 2023; Riordan-Short et al., 2023; Iverson et al., 2024). Here, I describe a new methodology for quantifying landscape connectivity in two dimensions.

Relatively few researchers measure landscape connectivity directly, as empirical studies sufficient to do so are difficult to conduct (Fagan and Calabrese, 2006; Ortega et al., 2023; Carroll et al., 2024; Morin et al., 2024), nearly impossible to replicate, and because the likelihood of observing interpatch movements will typically vary with local population sizes and demographic rates (Mcintire et al., 2007). Instead, mathematical models, computer algorithms, and movement simulations are frequently employed to obtain proxies for connectivity. Inferences used to be drawn from fragmentation indices, which are pattern metrics such as shape index, fractal dimension, or contagion (O’Neill et al., 1988; Turner, 1989). Fragmentation indices describe landscape patterns, and have been shown to have a limited ability to anticipate connectivity when the latter is inferred from the outcome of movement simulators (Schumaker, 1996). The methods used to evaluate connectivity have since changed considerably (Mestre and Silva, 2023), due largely to the development of tools that exploit graph and circuit theories (McRae, 2006; McRae and Beier, 2007; McRae et al., 2008; Urban et al., 2009; Perry et al., 2017). These studies, and others, have inferred patterns of animal movements across large landscapes (Carroll et al., 2012; Severns et al., 2013; Hromada et al., 2020; Finerty et al., 2023), identified strengths and weaknesses of protected area networks (Carroll et al., 2012), prioritized conservation and restoration activities (Dickson et al., 2017; Pither et al., 2023), and much more.

Graph theory is the study of mathematical objects called graphs. In this context, graphs are composed of nodes, which may represent quantities like resource patches, and edges, which always represent connections between pairs of nodes. Ecological models building upon graph theory (Urban et al., 2009), which I subsequently refer to as “graph models”, require access to a dispersal kernel (Fordham et al., 2014; Proença-Ferreira et al., 2023). Dispersal kernels are mathematical structures describing the likelihood of arrival at all possible future locations, conditioned on an object’s present location. Dispersal kernels can take the form of continuous probability density functions, but in graph theory they are typically square matrices. Once a dispersal kernel has been formulated, the mathematics of graph theory can be deployed to reveal a great deal about network or landscape connectivity (e.g., Perry et al., 2017). But the difficulty of collecting empirical movement data (Fagan and Calabrese, 2006) means that dispersal kernels are often derived from cost path estimates or similar measures (Fletcher et al., 2023). Unsurprisingly, conclusions drawn from pattern-based dispersal kernels can suffer from biological oversimplification (Fordham et al., 2014).

Circuitscape and Linkage Mapper (McRae et al., 2008, 2016), which I subsequently refer to as “circuit models”, have been adopted widely in ecology, conservation, and other disciplines (Dickson et al., 2019). Circuit models are software applications that use electrical theory to infer landscape connectivity from resistance surfaces (McRae, 2006; McRae and Beier, 2007; Pither et al., 2023). Resistance surfaces are raster maps in which every pixel has been assigned a value indicating how likely (low resistance) or unlikely (high resistance) an object under study would be to enter that cell. Resistance surfaces are often assembled from extensive empirical data sets describing gene flow across complex landscapes (Peterman, 2023; Calderón et al., 2024), or from extrapolations based upon movement information (Finerty et al., 2023). An advantage of resistance surfaces is their generality; these maps need not be species-specific, and the concept is extensible to the study of a wide variety of endpoints of interest (e.g. Tassi et al., 2015; Tarkhnishvili et al., 2016; Dickson et al., 2019; Buchholtz et al., 2023). A limitation stemming from the use of circuit models is that, regardless of the biological nuance embedded within a resistance surface, these tools have no direct way to account for dispersal ability or behavior.

Fragmentation indices measure structural connectivity. Graph and circuit models, in contrast, attempt to capture functional connectivity by shifting the perspective from landscapes to organisms (Carroll et al., 2012; Perry et al., 2017; Dickson et al., 2019; Finerty et al., 2023; Guarenghi et al., 2023; Pither et al., 2023). But the term functional connectivity spans a continuum of biological and behavioral realism that is not thoroughly represented by these models and methods (Drake et al., 2022). For a simple illustration of what is missing, imagine a landscape composed of an array of cells, each having a score indicating its quality. An individual occupying a cell scored one (poor quality) might readily elect to move into a cell scored three (moderate quality). But, for an individual occupying a cell scored five (optimal quality), this option may appear undesirable. Similarly, behaviors that affect movement distance and path tortuosity might be uniquely influenced by an individual’s perception of its recent movement history. When incorporated, this type of biological detail is likely to alter estimates of functional landscape connectivity.

Spatial population viability analysis (PVA) models are typically lifecycle simulators linked to landscape maps. Movement-only simulators are lower-complexity models that ignore much of the detail found in a PVA. But in spite of their relative simplicity, movement simulators can still incorporate dispersal ability, account for species-landscape interactions and disturbance, and capture behaviors in which future decisions are influenced by past experience. And simulation modeling has been widely used for evaluating functional landscape connectivity (e.g., Kramer-Schadt et al., 2004, 2011; Revilla et al., 2004; Revilla and Wiegand, 2008; Pe’er et al., 2011; Kanagaraj et al., 2013; Coulon et al., 2015; Diniz et al., 2020). Nevertheless, inferences about connectivity derived from movement models frequently rely upon visual inspections of cumulative dispersal traffic (Allen et al., 2016; Hauenstein et al., 2019; Day et al., 2020; Unnithan Kumar et al., 2022; Hofmann et al., 2023; Urbina et al., 2023), which my results (see below) suggest may be misleading. And we lack generic, reusable methods and tools that transform dispersal information (empirical or simulated) into utilitarian connectivity assessments complementing those obtained from graph or circuit models (but see Hofmann et al., 2023). My study attempts to address both limitations, and to provide readers with a general solution for teasing insights about functional landscape connectivity out of movement data, regardless of its source.

My methods are designed to draw conclusions about functional connectivity from movement data. Species’ vital rates and life cycles are not considered, and my results do not forecast population size, structure, extinction risk, or related measures (e.g., Hanski and Ovaskainen, 2000). And while I believe my methods will be useful for prioritizing mitigation and restoration, I have not coupled my workflow to a formal decision-making rubric (e.g., Westphal et al., 2003). I place my work within the context of graph and circuit models, but I do not make direct comparisons between these tools. With regards to circuit models, I instead emphasize the value of obtaining connectivity assessments that are sensitive to species’ movement ability and behavior. In the case of graph models, my methods do not constitute an alternative, but rather a means for obtaining biologically nuanced dispersal kernels. I do, however, make an implicit comparison within the context of simulation modeling by exploring the differences between maps of all individual movements versus those made strictly from paths connecting resource patches.

I begin by introducing my connectivity methodology, and then apply it to a simulated population of Fender’s blue butterflies (*Icaricia icarioides fenderi*) occupying a small portion of the species’ range. My focus is on illustrating the methods I have developed, and the Fender’s blue butterfly (FBB) case study is useful in this context (Mcintire et al., 2007). My FBB movement simulator was informed by data obtained from empirical studies (Schultz and Crone, 2001; Schultz et al., 2012; Cheryl Schultz, pers. comm.); and, to the extent possible, its design replicated that of *FendNet*, the original spatially-explicit and individual-based FBB movement simulator (Mcintire et al., 2007; McIntire et al., 2013; Severns et al., 2013).

## Materials and methods

### Software tools

**FIGURE 1:**
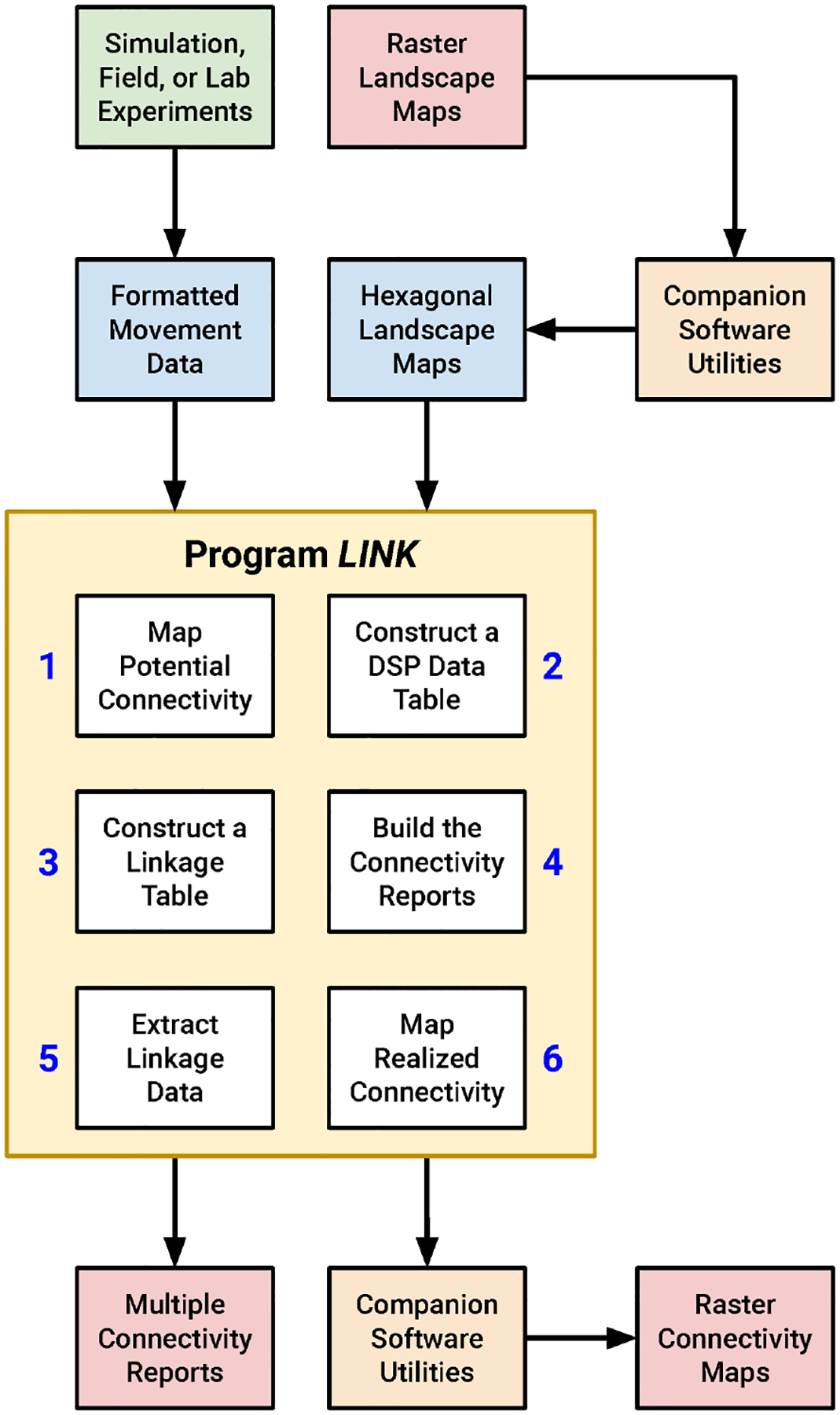
A diagram illustrating the proposed connectivity assessment workflow. LINK uses input movement data and landscape maps to generate a suite of connectivity reports and output maps. The process begins and ends with raster imagery, but the LINK program and some of its companion utilities work with hexagonally-tiled landscape maps. LINK itself contains six separate modules that each perform a portion of the overall connectivity analysis.

I wrote a C-language software utility that performs connectivity analysis, which I refer to below as “LINK”, since the program identifies resource patches linked by movement. I also developed a suite of complementary algorithms that simplify the processing of LINK input and output. Together, these applications can be assembled into a workflow for conducting rapid, actionable connectivity assessments (Figure 1). While the FBB movement simulations were conducted in HexSim (Schumaker and Brookes, 2018) all of my other analyses were performed using stand-alone utilities. I’ve made this code available to readers, along with a fully illustrated example connectivity analysis (see Supplementary Material). HexSim is a popular platform for developing spatially-explicit, individual-based life history simulators (Heinrichs et al., 2023; Lyons et al., 2023; Mims et al., 2023; Ransom et al., 2023; White et al., 2023).

The discussion that follows is, in large part, an exploration of the LINK utility and its potential for quantifying and visualizing landscape connectivity. That said, readers with some programming experience should be able to replicate, extend, and improve upon my methods and tools without reliance on HexSim or LINK. And those interested in quantifying connectivity in marine environments, forest canopies, or other spatially-complex systems could adapt my work to 3-dimensional landscapes.

### Study areas

The Fender’s blue butterfly case study runs within a map depicting a roughly 14,000 ha area situated in the approximate center of the species’ range. This map, which is made up from an array of hexagonal cells, was derived from an ASCII Grid file (https://en.wikipedia.org/wiki/Esri_grid) exported from a geographical information system by staff at the US Fish and Wildlife Service (USFWS). The Fender’s blue butterfly study area (Severns et al., 2013) is located in the Cardwell Hills, to the west of Corvallis, Oregon, USA. This site contains one of the largest extant populations of the species.

**FIGURE 2:**
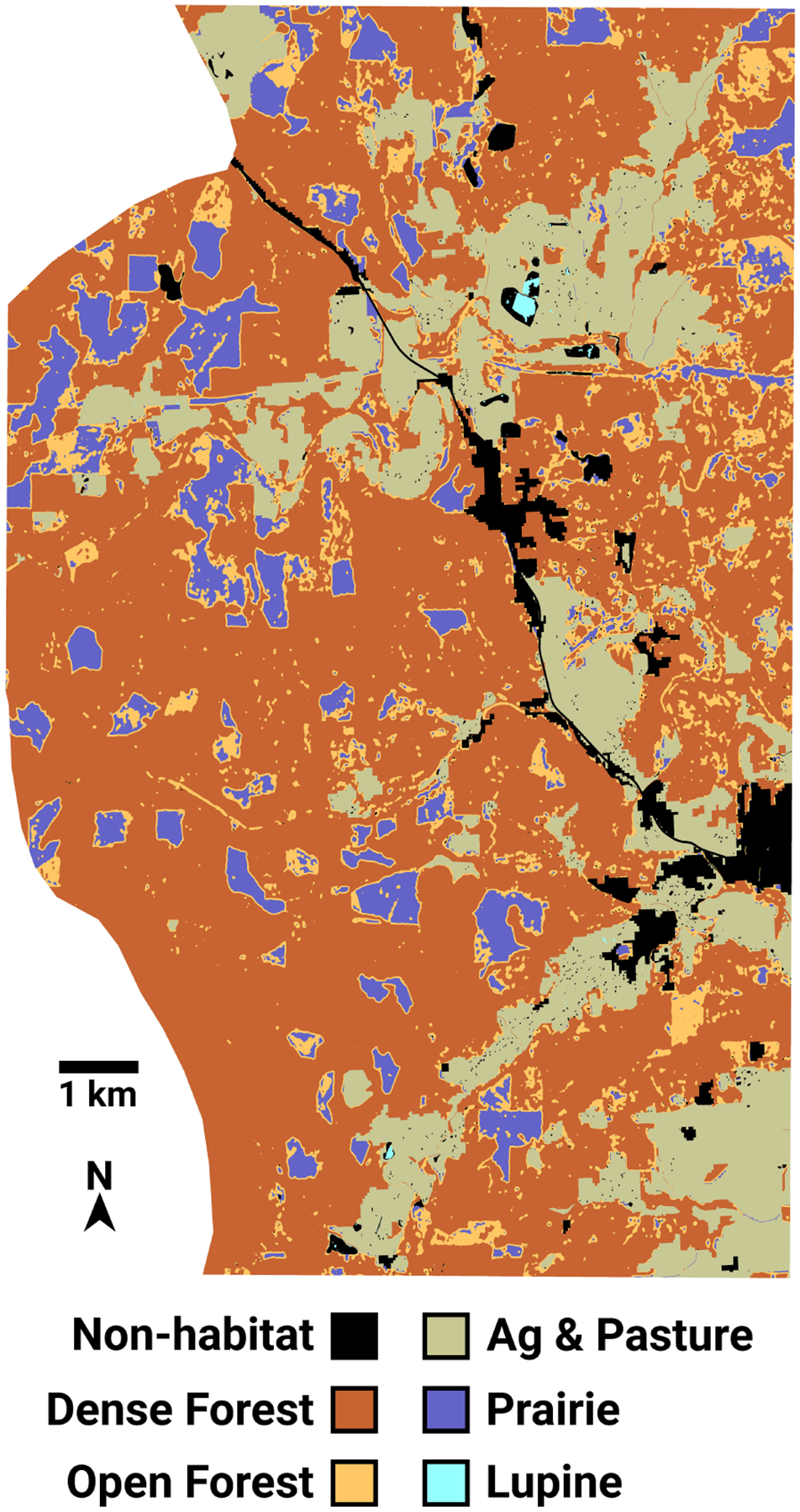
The Fender’s blue butterfly landscape, represented as a grid of 160 million hexagonal cells. Most lupine patches are too small to be resolved in the image.

The USFWS ASCII Grid file representing FBB land cover has an extent of 10,896 columns × 15,231 rows, with each pixel representing a square 0.836 m^2^ in size. This makes for a total landscape area of 13,876 ha. I resampled this raster image into a grid of hexagonal cells (a “hexmap”) containing 9962 columns and 16,081 rows, slightly in excess of 160M hexagons total. The width (measured between parallel sides) and area of each hexagon are 1.000 m and 0.866 m^2^. This fine-resolution map facilitated the simulation of individual FBB movements, which can be as short as three meters (see below). Each FBB land cover hexagon was assigned an integer score equal to the mode of the ASCII Grid pixels falling within that hexagon (Figure 2). Using a mode operator ensured that each hexagon was assigned an integer value, thus preserving the categorical nature of the input ASCII Grid file. Subsequently, 1639 ha that had been assigned a “no data” classification in the ASCII grid file were merged into its “non-habitat” category. Non-habitat is used as a generic designation for developed areas that FBBs will avoid. The resulting map of hexagonal cells contained six land cover categories (Table 1).

Kincaid’s lupine (*Lupinus oreganus*) is the sole larval host plant for the Fender’s blue butterfly (Liston et al., 1995; Schultz and Dlugosch, 1999; Schultz, 2001). FBB food resources are found within areas classified as lupine or prairie. FBBs can move about within all of the land cover types except for non-habitat, which they will not enter.

There are 140 distinct Kincaid’s lupine patches in the FBB map. Of these, 65 are completely isolated by non-habitat, which the simulated butterflies would not enter. (In reality, FBBs will occasionally move across small stretches of non-habitat, such as those attributable to roads. Our FBB habitat map, however, did not include a road network.) That left 75 accessible lupine patches that FBBs might potentially move between. These Kincaid’s lupine habitats comprise the focal patches for my butterfly connectivity analysis. The LINK program requires that unique IDs be assigned to every patch in its input patch maps. One of my utilities is designed to perform this labeling task (see Supplementary Material), and I used it to create a Kincaid’s lupine patch map suitable for use with LINK.

### Movement models

My movement models incorporate behavior, and thus their output stores information about functional landscape connectivity. The LINK utility, which I used to extract this information, imposes minimal constraints on model design. Specifically, a suitable model must (a) simulate movement as a sequence of discrete “steps” in which individuals move from a cell to one of its immediate neighbors, and (b) write out all individual movement records in a predefined format (see Supplementary Material). Importantly, the application of LINK need not be limited to modeling studies; my methods can also be used to analyze movement data collected in field or laboratory settings (Finerty et al., 2023).

Grids of hexagonal cells are ideal for simulating movement processes because, unlike square landscape tessellations, all neighbors are equidistant. Hence, HexSim, LINK, and some of the other programs referenced here are designed to work with arrays of hexagonal cells (a hexmap). Nevertheless, landscape data are almost always tiled using arrays of square pixels (a raster). For this reason, I supply readers with software utilities that convert traditional landscape maps into hexmaps, and that convert hexmaps back into raster imagery (see Supplementary Material). This suite of tools provides users with the convenience of beginning and ending with raster maps, while also eliminating artifacts that can accompany the use of such data in movement simulations.

My FBB movement model grew out of a series of conversations with species expert Dr. Cheryl Schultz, who had gathered empirical data describing Fender’s blue butterfly turning angles and path lengths as a function of land cover type, and is a co-developer of the *FendNet* model. All simulated FBBs made a series of 250 separate movements, which approximates an actual butterfly’s search effort over their single-season lifetime. Each movement step was characterized by a path length (number of steps), autocorrelation (turning angle), and a probability of moving directly towards the species’ host plant (Schultz and Crone, 2001), Kincaid’s lupine, referred to subsequently as “lupine”. In order to match the empirical information, the values used for these parameters were adjusted depending on the land cover class that each butterfly occupied at the time a movement was initiated (Table 2).

In advance of each of the 250 separate movement events, every simulated FBB was placed into one of two behavior classes. Those not already located within lupine evaluated whether they were within 50 meters of a lupine patch. If so, these FBBs used a “go to lupine” probability to determine whether they should move directly towards lupine. FBBs presently within a lupine patch, those nearby who elect not to move directly towards lupine, and butterflies situated far from lupine all moved semi-randomly. Movement path lengths were imposed regardless of behavior class, but autocorrelation only influenced the behavior of butterflies moving semi-randomly. FBBs moving directly towards lupine always took the most efficient path available to them. Butterflies moving semi-randomly blended a correlated random walk with limited emergent taxis towards more “desirable” (Cheryl Schults, pers. comm.) land cover types (Table 2).

Both the HexSim FBB model and FendNet were designed using the same empirical data sets and subsequent analysis (Schultz et al., 2012). The most significant differences between the two are that FendNet is a full lifecycle model developed in *SELES* (Fall and Fall, 2001) for which movement behavior is in part expressed via resource-specific turning angles. My model, in contrast, only simulates movement, and it uses autocorrelation rates rather than turning angles. I developed a relationship linking turning angles to autocorrelation rates that facilitated this conversion. The FendNet model was validated using data from a study area 80 km to the south of the Cardwell Hills site (Mcintire et al., 2007). This prior assessment suggests the HexSim simulator is likely to be a reasonable proxy for movement in the Cardwell Hills system.

Because my goal was to evaluate inter-patch connectivity, introducing FBBs into the interior of lupine patches was computationally inefficient. Thus, I initially placed butterflies into every lupine patch edge, excepting those hexagons bordered strictly by lupine and/or non-habitat. Given this criteria, 2797 lupine hexagons qualified as valid starting locations. I ran 1000 model replicates, thus simulating roughly 2.8M butterflies, and generating 0.7B distinct movement records, each varying in length between 3 and 11 hexagons.

### Connectivity metrics

The LINK utility ignores movement steps that precede an individual’s arrival at its first focal patch. For that reason, I initially placed all simulated individuals into focal patches. LINK begins by aggregating all of the movement records associated with a specific individual into a single continuous movement path. This was somewhat involved for the simulated FBBs, who each moved 250 times in a randomized order, meaning that individual movement records were scattered throughout >200 gigabytes of model output. LINK next measures the rates at which individuals move between focal patches. To do so, it breaks each aggregate movement path into “connecting segments” that begin and end in separate focal patches. Movement steps in the interior of focal patches are not included in connecting segments, but LINK separately records the frequency with which individuals (a) begin and end in the same focal patch, and (b) begin in a focal patch but end in a different land cover type. LINK uses this information when it constructs dispersal kernels that capture the probability of moving between every pair of focal patches.

LINK also constructs a pair of maps that illustrate (a) “potential connectivity”, defined as the cumulative number of times each hexagon was visited, and (b) “realized connectivity”, which only records visitations associated with connecting segments. Potential connectivity may be thought of as an inverted emergent resistance surface (high potential equaling low resistance) that conflates absence and avoidance. In contrast, realized connectivity serves as a visual representation of a dispersal kernel. Finally, LINK uses the connecting segments to construct a report describing “connectivity clusters”, defined as collections of focal patches linked by movement. This report includes values for “cluster traffic”, the number of focal patches per cluster, and the IDs of every patch making up each cluster. A cluster’s traffic is defined as the number of connecting segments linking all of the focal patches it contains.

While developed independently, the initial stages of my connectivity analysis are reminiscent of a portion of the methodology published by Hofmann et al. (2023). That being the case, our approaches for drawing conclusions about functional landscape connectivity are distinct yet complementary. Hofmann et al. (2023) and others (e.g. Carroll et al., 2012; Sarker et al., 2019), use a graph theory metric termed “betweenness centrality” to infer connectivity from collections of simulated movement paths. In contrast, my strategy involves isolating the portions of movement paths that link resource patches, and using this information to identify and interrogate connectivity clusters.

## Results

**FIGURE 3:**
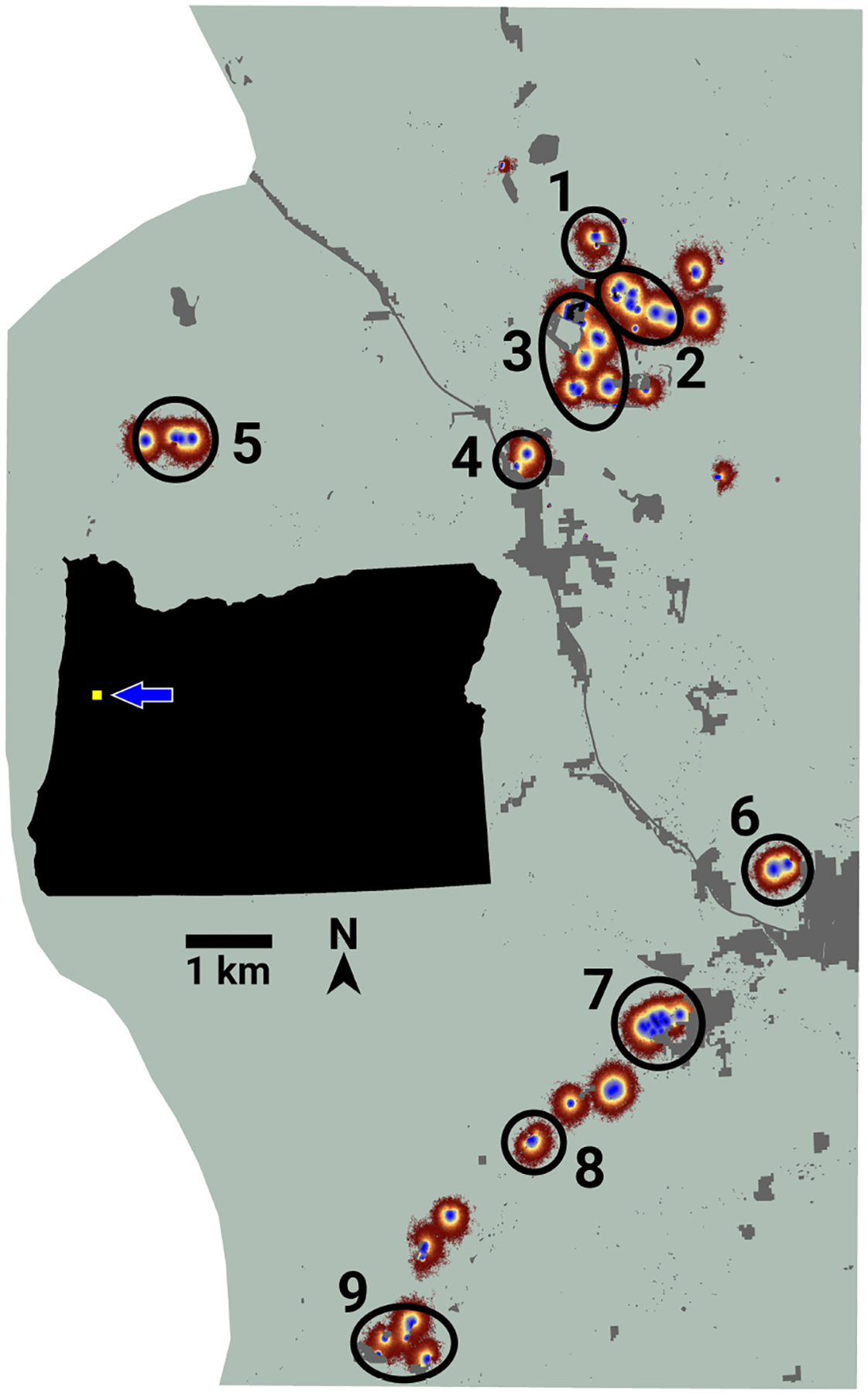
An image of potential connectivity for the Fender’s blue butterfly system. Numbered ovals indicate the approximate locations of nine emergent connectivity clusters. The inset map shows the study area’s location relative to the state of Oregon, USA.

LINK’s analysis of the FBB movement data revealed the presence of nine separate connectivity clusters (Figure 3), which ranged in size from 3 to 13 lupine patches. Cluster area and traffic varied across three and four orders of magnitude, respectively (Table 3). Of the 75 accessible lupine patches, 59 were included in the nine connectivity clusters. The remaining 16 accessible patches were functionally disconnected.

**FIGURE 4:**
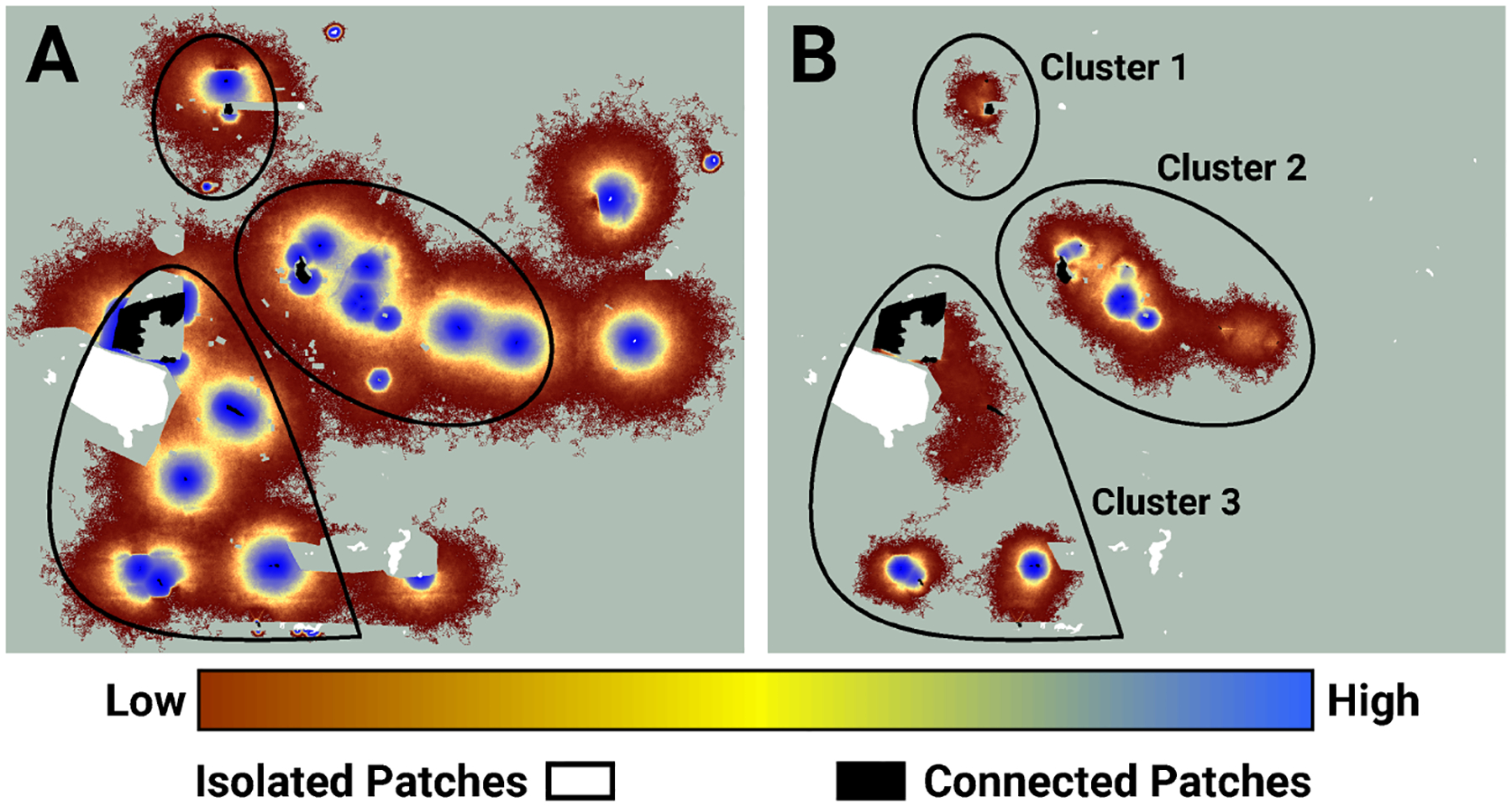
An image of potential connectivity in the vicinity of clusters 1, 2, and 3 **(A)**. The image of realized connectivity used to identify the three clusters **(B)**. The relative values of both potential and realized connectivity are indicated by the colorbar. Non-habitat and areas unused by FBBs are shown in light green. The black outlines indicate the approximate cluster locations. Isolated (white) and connected (black) lupine patches have been superimposed on the images. Some lupine patches may be too small to resolve. Each panel is 2.2 km in width.

I used LINK’s maps of potential and realized connectivity to more closely examine clusters 1–3, 7–8, and 9 (Figure 3). Based on potential connectivity, almost all of the lupine patches in the vicinity of clusters 1–3 would appear to be part of a single expansive “supercluster”. In contrast, the map of realized connectivity (Figure 4), and LINK’s cluster analysis, suggest that functional connectivity is limited in this region. A direct comparison of the two connectivity maps (Figure 4) indicates there is a possibility of reconnecting clusters 2 and 3, presumably via habitat restoration, as a large number of simulated butterflies explored the intervening landscape. In contrast, many fewer butterflies moved within the gap separating clusters 1 and 2. These results also suggest that cluster 2 might be extended to the east. Finally, the realized connectivity data suggests that cluster 3 itself is only tenuously connected, and likely vulnerable to future habitat loss.

**FIGURE 5:**
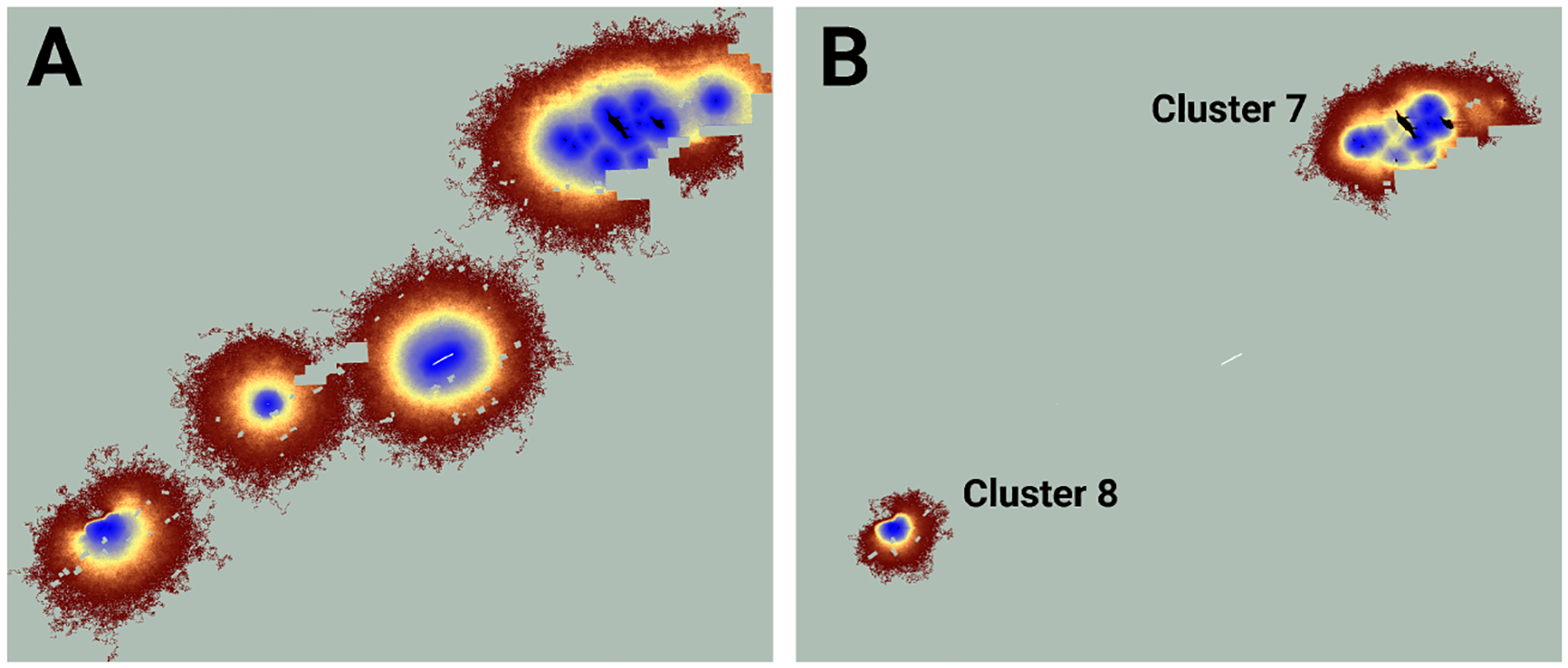
An image of potential connectivity in the vicinity of clusters 7 and 8 **(A)**. The image of realized connectivity used to identify the two clusters **(B)**. Isolated (white) and connected (black) lupine patches have been superimposed on the images. Some lupine patches may be too small to resolve. Each panel is 2.2 km in width. See Figure 4 for additional color-related details.

**FIGURE 6:**
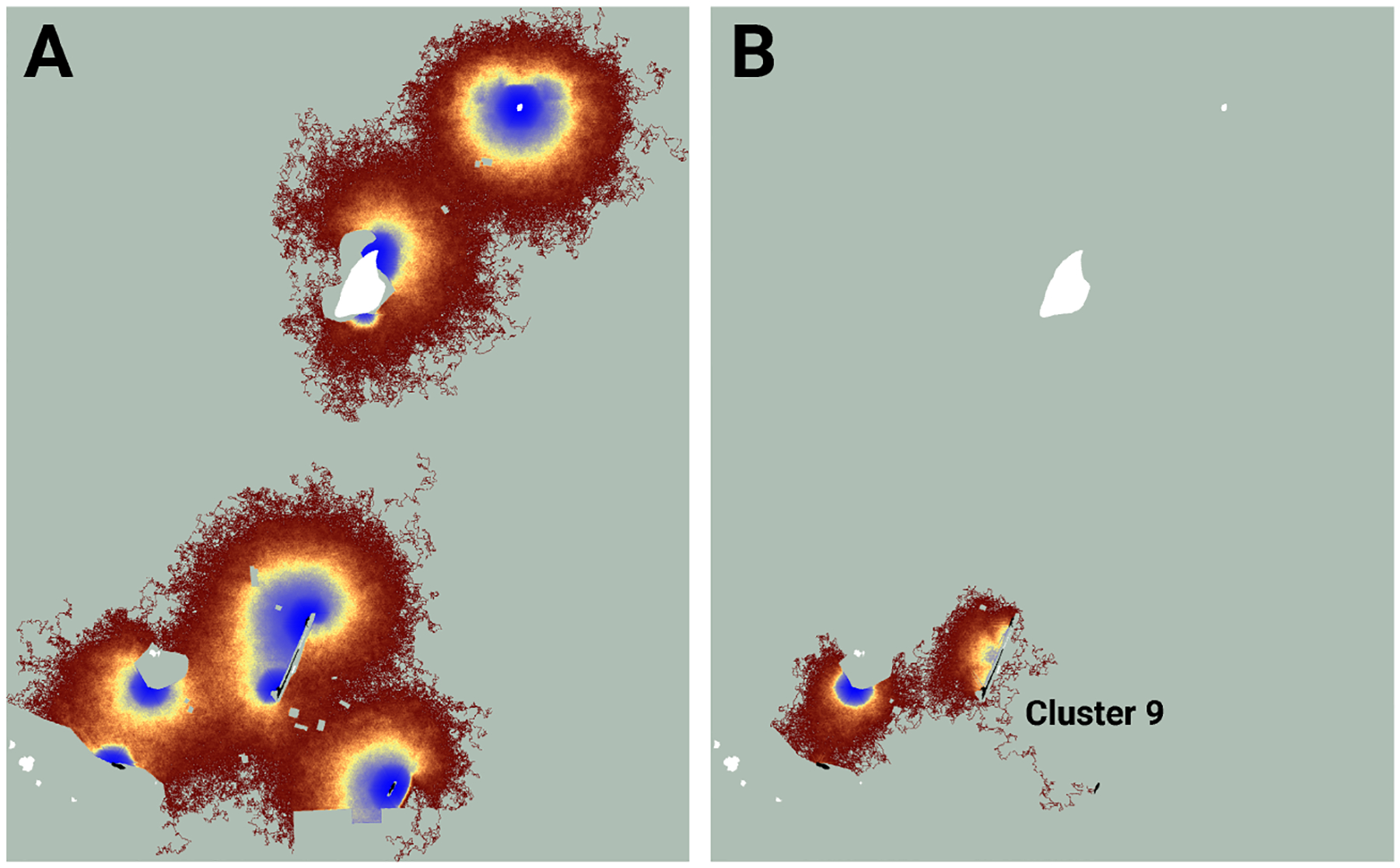
An image of potential connectivity in the vicinity of cluster 9 **(A)**. The image of realized connectivity used to identify the cluster **(B)**. Isolated (white) and connected (black) lupine patches have been superimposed on the images. Some lupine patches may be too small to resolve. Each panel is 1.4 km in width. See Figure 4 for additional color-related details.

Similarly, while the map of realized connectivity suggests that clusters 7 and 8 are functionally distinct, the map of potential connectivity indicates that the possibility exists to tie this entire area into a single connected supercluster (Figure 5). Given that clusters 7 and 8 exhibited the system’s largest cluster traffic (Table 3), the relative benefit of targeting this area for restoration may be high. A parallel inspection of cluster 9 (Figure 6) suggests that restoration activities in the immediate vicinity of the existing functionally connected lupine patches might benefit the FBB population; but the creation of a robust new supercluster here may require substantial investment.

**FIGURE 7:**
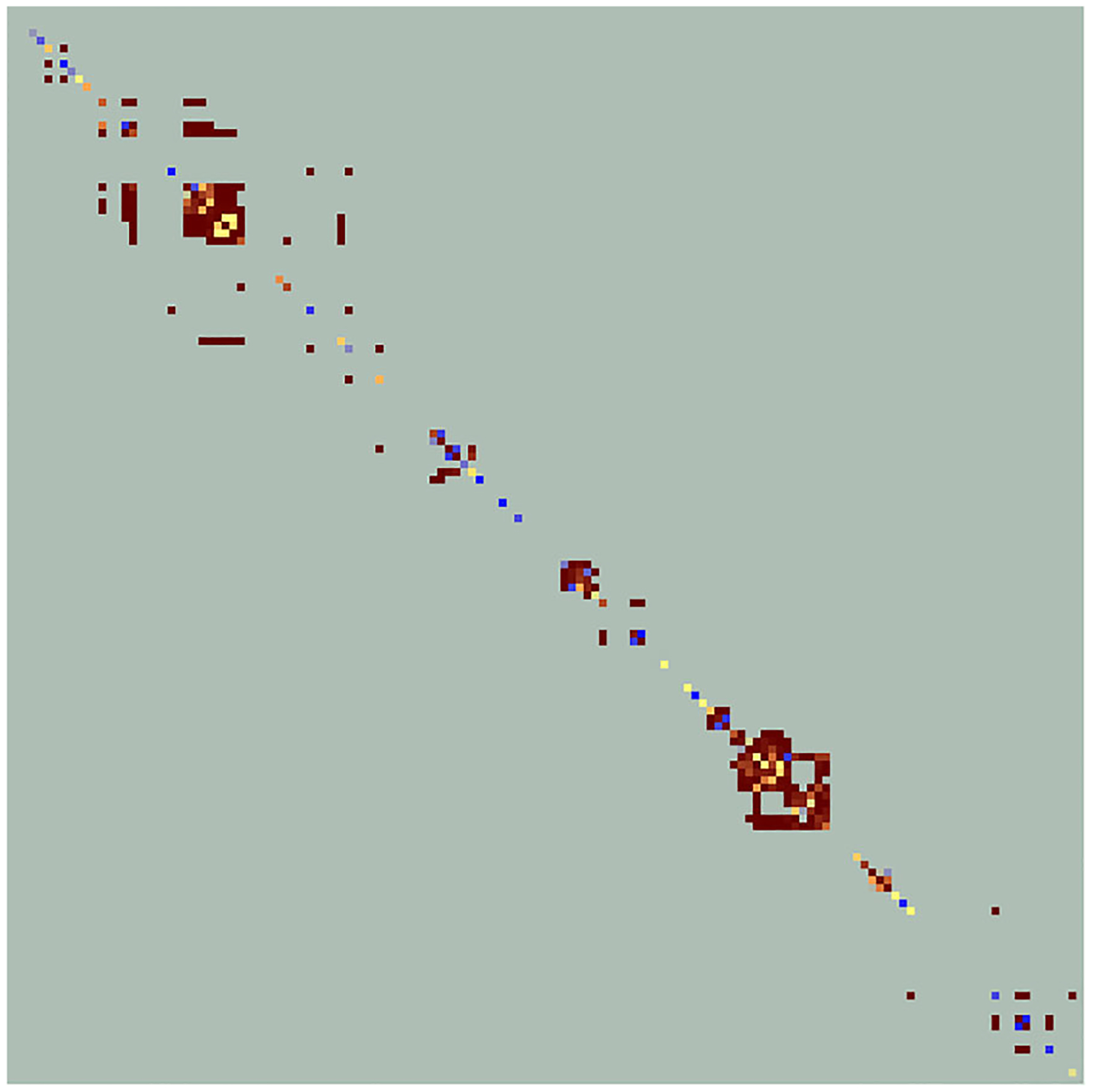
A heat map analog of the emergent FBB dispersal kernel, which is 140 × 140 lupine patches in size. Zero-valued cells are displayed in light green. Cells falling along the diagonal indicate movement rates from a patch to itself. Asymmetries imply unequal directional rates of exchange between pairs of lupine patches. Connectivity cluster size is correlated with the number of off-diagonal cells lying within specific portions of the image. See Figure 4 for additional color-related details.

LINK generated an emergent FBB dispersal kernel in the form of a sparse square matrix with dimension 140 (the number of lupine patches) containing 19,600 cells. This matrix, which is best imaged as a heat map due to its size (Figure 7), has values that range between zero and 0.996. The value of the cell in column *i* and row *k* represents the probability that a FBB located in lupine patch *i* would subsequently move to patch *k*. The sum of column *i* equals the probability that a butterfly located in patch *i* would move to any lupine patch (including itself), while 1.0 minus this quantity is the likelihood that a FBB leaving that location would stop moving somewhere in the non-lupine matrix. The sum of row *k* represents the probability that a butterfly would travel to patch *k* from any other lupine patch, including itself.

## Discussion

Researchers commonly use graph theory, circuit models, and spatial simulators to quantify landscape connectivity. Models incorporating graph theory (e.g. Bastian et al., 2009; Foltête et al., 2012) use putative dispersal kernels to assess the importance of network “nodes” and “edges”. But by necessity, dispersal kernels are often derived from landscape geometry rather than movement data (Dickson et al., 2019; Finerty et al., 2023); in such cases, conclusions drawn from these models can lack realism (Fordham et al., 2014). And even when sufficient movement data are available, it can prove difficult to extract a dispersal kernel from this information. The Circuitscape family of tools (McRae et al., 2008, 2016) infer patterns of landscape connectivity from simulations of electrical current flowing across resistance surfaces. But current flow cannot capture movement behavior, and will only fall to zero where resistance is infinite. In contrast, movement simulators can replicate complex individual behaviors, and their estimates of movement rates may drop to zero in any location due to energetic constraints, perceived threats, and so on. Unfortunately, generic, flexible tools that can convert movement data (simulated or real) into connectivity assessments have not been available, forcing researchers to develop independent solutions on an as-needed basis.

Here, I have introduced a general methodology for extracting dispersal kernels from movement data. LINK’s emergent dispersal kernels, which retain the biological detail captured within real or simulated movement data, can be substituted into existing graph models, thus increasing their realism. My software can also generate assessments of functional landscape connectivity directly from any properly-formatted movement dataset. LINK’s illustrations of potential connectivity are reminiscent of the current flows obtained from circuit models, and of the maps of cumulative individual movement paths derived from simulation modeling experiments. But the considerable differences between LINK’s potential and realized connectivity maps highlight the utility of discriminating between all landscape locations that have been visited collectively, versus just the sites that were traversed during successful movements between resource patches. Policy recommendations informed by the former are likely to differ significantly from those influenced by the latter.

I used the Fender’s blue butterfly case study to explore the differences between potential and realized connectivity, and to illustrate how maps of these quantities might be useful for ranking management strategies. Simulated FBBs frequently proved unable to move between lupine patches that exhibited high potential connectivity. For example, maps of potential connectivity suggest the lupine patches in the vicinity of connectivity clusters 1, 2, and 3 might be a low priority for habitat restoration (Figure 4). The results from my evaluation of realized connectivity indicate exactly the opposite. Similar mismatches arose in the neighborhood of connectivity clusters 7 and 8 (Figure 5) and cluster 9 (Figure 6); they are likely ubiquitous across the FBB system.

Data is not currently available to directly test the validity of LINK’s FBB connectivity assessments. The most similar existing connectivity analysis is substantially different, and was conducted in a separate portion of the species’ range (Mcintire et al., 2007). While a great deal of information has been gathered on FBB movement in various portions of the species’ range (e.g., Schultz et al., 2012), the only other Cardwell Hills connectivity study (Severns et al., 2013) produced findings that, by design, cannot be compared to LINK’s output. The results from the present study should therefore be approached as hypotheses to be challenged and refined. More generally, the methodology I’ve described here will benefit from future applications involving other ecological systems, landscapes, and life histories.

## Conclusions

Graph- and circuit-based connectivity assessments are compelling and influential, but they frequently incorporate little biological nuance (Drake et al., 2022). In contrast, spatial PVAs have been trending towards realism and defensibility (D’Elia et al., 2022; Pili et al., 2022; Heinrichs et al., 2023), though this has been accompanied by increasing development time and effort (e.g. Snyder et al., 2019). Movement-only simulators provide a compromise; they can capture sophisticated individual behaviors (Schultz and Crone, 2001; Brown et al., 2017), species-landscape interactions, and disturbance, while still being parsimonious and quick to assemble. And the data generated by these models are uniquely well-suited for catalyzing new insights into functional landscape connectivity. But researchers lack generic methods for inferring functional connectivity from simulation model output, and thus frequently end up developing study-specific software and algorithms that are not readily transferable to others. Here, I provide a general solution constructed with reuse in mind.

My LINK utility is designed to extract connectivity metrics from movement data regardless of how this information was obtained. The analyses I’ve described involve five steps: (1) designing a movement model and running simulations, (2) processing the simulation output, (3) generating a dispersal kernel, (4) mapping and visually inspecting both potential and realized connectivity, and (5) performing a connectivity cluster analysis. LINK automates steps 2–5, thus greatly simplifying the workflow. Though I used HexSim for simulating FBB movement, all my other pre- and post-processing steps were performed by stand-alone software utilities. To facilitate the transfer of this technology, I have provided a worked example of the entire process, beginning with a land cover map and ending with a full connectivity analysis (see Supplementary Material). This illustration does not require the use of HexSim, thus minimizing the investment required to replicate and improve upon my methods.

By isolating Fender’s blue butterfly movements that join distinct lupine patches, I was able to identify connectivity clusters, and quantify rates of movement between cluster patches. My visual comparisons of potential versus realized connectivity suggest where restoration efforts might most effectively enhance landscape connectivity, and can help identify resources at risk of becoming functionally disconnected. Additionally, LINK’s emergent dispersal kernels should facilitate the application of graph-theoretic models to conservation challenges set within complex landscapes. Once a dispersal kernel has been obtained, the remaining components of a graph model are relatively straightforward to assemble. LINK’s results also have the potential to simplify future PVA model development. For example, smaller more computationally efficient predictive models could be developed separately for each of LINK’s emergent connectivity clusters. And these new focal-area PVAs would no longer need to simulate movement, as they could instead use a pre-computed dispersal kernel.

## Supplementary Material

Supplement1

## Figures and Tables

**TABLE 1:** Land cover types and areas from the Fender’s blue butterfly hexmap.

Land Cover Type	Hexagons	Hectares
Non-habitat	23,847,287	2066
Dense forest	85,329,621	7390
Open forest	15,565,730	1348
Ag. and pasture	24,738,745	2143
Prairie	10,593,780	918
Kincaid’s lupine	123,759	11
TOTAL	160,198,922	13,876

The raster ASCII Grid input file contained the same six land cover classes, plus a separate no-data class. When the hexmap was constructed, the no-data and non-habitat classes were merged.

**TABLE 2:** HexSim FBB movement model parameters.

Land Cover Type	Path Length (meters)	Autocorrelation (percent)	Go to Lupine (probability)
Non-habitat	Unused by simulated Fender’s blue butterflies		
Dense forest	7	68	0.75
Open forest	7	68	0.75
Ag. and pasture	11	71	0.25
Prairie	11	74	0.25
Kincaid’s lupine	3	35	0

The land cover classes have been sorted based on their anticipated desirability for the butterflies.

**TABLE 3:** The LINK connectivity cluster analysis for the FBB model.

	Cluster Traffic	Lupine Patches	Cluster Area
Cluster 1	349	3	929
Cluster 2	346,486	13	2591
Cluster 3	496,290	10	25,818
Cluster 4	248,465	3	45
Cluster 5	368,602	5	943
Cluster 6	331,611	3	168
Cluster 7	1,025,837	13	2909
Cluster 8	540,085	3	50
Cluster 9	66,593	6	947

Lists of individual patch IDs have been replaced with cluster area, measured as the total number of lupine patch hexagons. LINK assigns cluster IDs in map order, from the upper-left to lower-right.

## Data Availability

The original contributions presented in the study are included in the article/Supplementary Material. Further inquiries can be directed to the corresponding author.
