# Supplementary material for: A rapid assessment methodology for quantifying and visualizing functional landscape connectivity
Source: Front Conserv Sci. Author manuscript; Available in PMC 2025 Jul 4. (PMC11457150; doi:10.3389/fcosc.2024.1412888)
Supplement: Supplement1 [file NIHMS2010488-supplement-Supplement1.zip › Supplemental Information/Supplemental Information.pdf]

Supplementary Material

A. Background

This document walks readers through an example connectivity assessment. References will be made to the *HexSim* simulation modeling environment. *HexSim* is a free application that readers may download from www.hexsim.net. I also discuss my LINK utility, and several companion applications. LINK is the principal software I use to conduct connectivity assessments. The remaining applications play a role in pre- and post-processing.

Many of the tasks below involve two additional software packages. *XnView* (www.xnview.com) is a platform for viewing and manipulating raster imagery. I use the "Classic" version of *XnView* to visualize maps, and to convert images from a Sun Raster format (https://en.wikipedia.org/wiki/Sun_Raster) into PNG files. *Cygwin* (www.cygwin.com) is a unix operating system that runs on Windows. I use *Cygwin* to compile and run the software utilities I've written. Both *XnView* and *Cygwin* are free. Note that alternatives to *XnView* and *Cygwin* are available; these are just the applications I am the most familiar with.

When installing *Cygwin*, users will want to be sure to include the C-language packages. Once that has been done, my utilities can be compiled by typing "cc <utility>.c", where <utility> is replaced by the name of the program. I will often leave off the ".c" and ".exe" extensions when referring to my software utilities.

Below, I include reproductions of *Cygwin* input and output. This content will be displayed as follows: `CMD-#    >>  utility  parameters`. Here, ">>" represents the *Cygwin* prompt. All of my utilities should generate a usage statement when run in *Cygwin* without any command-line arguments. In the content below, I will assume readers have already compiled the utilities.

Many of my programs require the specification of input or output "hexmaps", which are just maps made up from collections of hexagonal cells. Users will frequently need to supply the hexmap geometry by entering the number of rows, plus the minimum and maximum number of columns per row. Hexmaps can be "wide" (all rows contain the same number of hexagons), or "narrow" (even rows are one hexagon shorter).

B. Obtaining the Example Data and Utilities

Prior to using LINK, readers must first obtain the requisite input and software utilities. These can all be found within the zip file from which this document was extracted. Readers will need each of the following files:

• Habitat.asc	A fabricated map of integer resource patches.
• asc2csv.c	Builds a hexmap from an ASCII Grid file.
• asc2sun.c	Builds a Sun Raster from an ASCII Grid file.
• assign_patch_id.c	Converts a hexmap into a patch map.
• bin_hexmap.c	Adds contrast to the connectivity hexmaps.
• csv2png.py	Builds a PNG image from a CSV hexmap.
• csv2sun.c	Builds a Sun Raster from a CSV hexmap.
• LINK.c	Performs connectivity assessments.
• randomize_patches.c	Randomizes the IDs of patch map patches.
• run_movement.c	Simulates movement within a hexmap.
• scan_ascii_grid.c	Quantifies the content of an ASCII Grid file.

The first item above is a fabricated habitat map that we'll use as input to the program named *run_movement*. We'll also use the habitat map to construct a patch map, since patch maps are required by the LINK utility. Before we can proceed with *run_movement* or patch map construction, we must first convert the Habitat.asc into a hexmap.

The remaining items in the above list are software utilities. The download includes C-language utilities, plus one python program (an alternative to *csv2sun.c*). I find *csv2sun.c* easier to use than *csv2png.py*. But, *csv2png.py* is necessary for imaging extremely large hexmaps, as there is apparently an upper limit on image size associated with Sun Raster files. I ran into this limit when constructing manuscript figures 2 and 3.

A table of contents for the remainder of this document is provided in Table S1.

Table S1. A Table of Contents for sections C, D, and E.

SECTION	PAGE	TITLE
C1	3	Examining the Input Habitat Map
C2	5	Building a Hexmap from the Input Habitat Map
C3	7	Visualizing the Habitat Hexmap
C4	8	Constructing a Patch Map
C5	11	Simulating Movement
D1	14	Mapping Potential Connectivity
D2	16	Building a DSP Data Table
D3	18	Constructing a Linkage Table
D4	19	Assembling the Connectivity Reports
D5	21	Extracting Connectivity Data
D6	22	Visualizing Realized Connectivity
E	26	Concluding Thoughts

C. Building Hexmaps and Simulating Movement

This section takes readers through the process of assembling the input data required by the LINK utility.

Step C1. Examining the Input Habitat Map

The download described in Part B, above, includes a fabricated habitat map in ASCII Grid format. Here, we begin by interrogating and visualizing this map.

First, we'll use the *scan_ascii_grid* utility to gather some basic information about Habitat.asc. This utility's output will differ depending on whether the input map contains integer or floating point data. A reproduction of the *Cygwin* command and output is shown below.

```
CMD-01:    >> scan_ascii_grid Habitat.asc

           This Image Contains Integer Data

           Image Header Information

                   Nbr Cols = 2500
                   Nbr Rows = 2500
                   Non-Data = -9999
                   Data Max = 9

           Image Histogram

                   No-Data Pixels: 0

                   Category 0: 2209594
                   Category 1: 1025692
                   Category 2: 713918
                   Category 3: 529986
                   Category 4: 434036
                   Category 5: 322740
                   Category 6: 307128
                   Category 7: 261446
                   Category 8: 222306
                   Category 9: 223154
```

Second, we'll create an image of the ASCII Grid file. To do this, issue the *Cygwin* statement:

```
CMD-02:    >> asc2sun Habitat.asc Habitat_Raster.sun 1
```

In the above command, Habitat.asc and Habitat_Raster.sun are the input and output filenames. The 1 at the end of the command line tells the utility to use a blue-yellow-red colormap. Low values will be displayed in blue, intermediate values in yellow, and high values in red. The other valid colormap option is 0, which produces an image that trends from red to yellow to blue.

Now, we can use *XnView* to visualize the Sun Raster image, and convert it into a PNG file (Fig. S1, left).

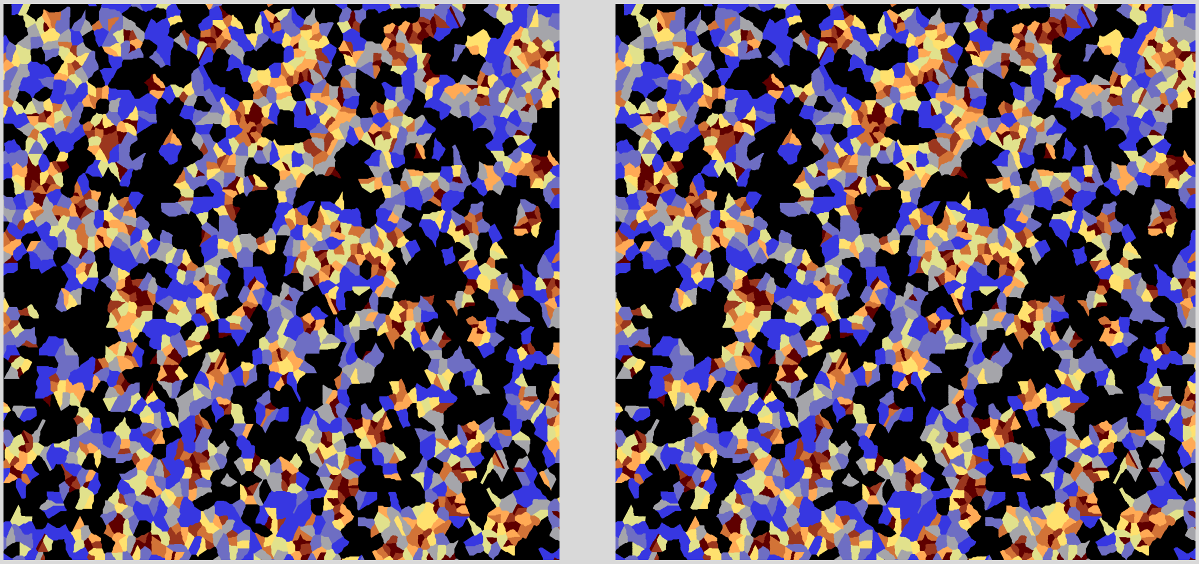

Figure S1. Images generated from Habitat.asc (left) and Habitat.csv (right). Slight differences will become apparent at high resolutions.

Step C2. Building a Hexmap from the Input Habitat Map

I use the term "hexmap" to refer to a map composed of hexagonal cells. Hexmaps may be stored as a CSV file containing two data columns. The first column will record hexagon IDs, and the second column lists hexagon scores. CSV hexmaps will frequently contain a one-line header, and my utilities that read hexmaps will always expect a single-line header has been included.

HexSim can import CSV hexmaps, and can also create a hexmap from an ASCII Grid file; see the *HexSim* user's guide for more information. My *asc2csv* utility can also build a CSV hexmap from an ASCII Grid file. To list off the parameters needed by *asc2csv*, invoke the utility without any command-line arguments:

```
CMD-03:    >> asc2csv

              Usage:  asc2csv  input_file  output_file
                      data_type=[int|float]  analysis=[mean|mode|binary]
                      sampling_effort=[low|medium|high]
                      hex_width rows min_cols max_cols
```

The conversion of an ASCII Grid image into a hexmap involves sampling. Specifically, a grid of hexagonal cells is superimposed upon the raster image, and each hexagon is assigned a score based on the values of the pixels it contains. The *asc2csv* utility provides three sampling resolutions: low, medium, and high. In this example, I use the medium resolution. The utility also provides three analysis options: mean, mode, and binary. Here, I'll use the mode.

The *asc2csv* utility can compute missing grid extent parameters. Users must request this be done by supplying a zero on the command-line for any grid extent attribute they'd like the program to compute. Note that at least one of the four extent parameters must be specified. In this example (see below), I'll ask the utility to build a grid using hexagons that are 1 meter wide, and let it figure out the rest on its own. This will produce a hexmap with 2866 rows and 2499 columns (min & max). Note that, because my hexagons are oriented with a point facing up, the number of columns can be fixed, or it can vary by one hexagon in every other row.

A reproduction of my *Cygwin* command is displayed in the image below.

```
CMD-04:    >> asc2csv Habitat.asc Habitat.csv int mode medium 1 0 0 0

              Reading Input File

              Rows, Min_Cols, Max_Cols : 2886, 2499, 2499
              Hex Width & Area (m, ha) : 1.000000, 0.000087

              Generating Hexmap: 100% Done...
              Saving Output File
```

The command line arguments I used above can be interpreted as follows:

- Habitat.asc. This specifies the input map, which must be an ASCII Grid.
- Habitat.csv. This is the name to be assigned to the output hexmap.
- Data Type = "int". This indicates that Habitat.asc contains integer data.
- Analysis = "mode". This tells the utility to assign hexagon scores using a mode operator. Each hexagon circumscribes a number of ASCII Grid pixels, and will be assigned a score equal to the mode of those integer pixel values.

- Sampling Effort = “medium”. The number of samples per hexagon can be 1760 (*low* effort), 7040 (*medium* effort), or 27,984 (*high* effort). Larger sample sizes provide more exacting estimates of each hexagon’s “true” value. The code will run more quickly when fewer samples are taken.
- Hex_Width = “1”. Habitat.asc has a header specifying that each pixel is 1 meter across, and thus represents a 1 m^2 area. Here, I am asking the utility to set the hexagon width equal to the pixel width. Any value could have been used, but this parameter must be supplied in the same units as the ASCII Grid file header, which by definition is meters.
- Grid Geometry. I have set the rows, min_cols, and max_cols values to zero. This makes the utility determine those quantities on its own. The algorithm will assign values that make the hexmap fill as much of the ASCII Grid map as possible, without exceeding the image boundaries.

Step C3. Visualizing the Habitat Hexmap

An easy way to examine the hexmap created in the previous step is to convert it into a raster image. *HexSim* can import and display a hexmap in CSV format, and it can export hexmaps as raster images. See the *HexSim* user’s guide for background and instructions. Here, we will describe the use of *csv2sun.c*, and alternatively *csv2png.py*, as a substitute for *HexSim*.

The utility *csv2sun*, is a C-language program that converts a hexmap in CSV format into a Sun Raster. *XnView* (discussed above) can subsequently convert a Sun Raster file into a more common image type, such as PNG. The *csv2png* utility is a python analog that directly constructs a PNG image. These utilities require the following information:

- Input filename
- Output filename
- Image resolution (small, medium, large)
- Colormap (0 or 1)
- Rows, min_cols, max_cols

When using *csv2sun*, this information must be passed on the command line. When using *csv2png*, the inputs are typed directly into the program source code. Image resolution is specified using [s | small], [m | medium], or [l | large]. Setting the colormap to 0 produces an image transitioning from red to yellow to

blue. Setting the colormap to 1 creates a colormap that transitions from blue to yellow to red.

I find *csv2sun.c* more convenient than *csv2png.py*. However, there is apparently an upper limit to the size of valid Sun Raster images, making *csv2png* necessary for use with extremely large hexmaps. Having a C-language version of *csv2png* would be useful, but I have not successfully written such a program.

To begin, let's first run *csv2sun* with no arguments, in order to obtain the command line options:

```
CMD-05:    >> csv2sun

              Usage:  csv2sun  hexmap  output  size  colormap
                      rows  min_cols  max_cols
```

Next, we'll create an image by issuing the following command:

```
CMD-06:    >> csv2sun Habitat.csv Habitat_Hexmap.sun m 1 2886 2499 2499
```

This makes the utility construct a medium-resolution image from the hexmap using a blue-yellow-red colormap. *XnView* can subsequently be used to visualize the image and convert it into a PNG file (Fig. S1, right).

At low resolution, the image should look identical to that obtained from the ASCII Grid file. The two images are, in fact, slightly different. The image derived from Habitat.asc is a raster composed of square pixels, while this new image is a hexmap composed of hexagonal cells. Also, the two images' aspect ratios are not identical.

Step C4. Constructing a Patch Map

In order for LINK to complete its connectivity analysis, the utility will require access to a patch map. This is because patch maps allow LINK to identify portions of movement paths that connect discrete resource patches. Patch maps will typically be derived from the spatial data being used to simulate movement, but this is not required; the patch map and other imagery being used need not share any features whatsoever. There are two steps to patch map construction. First, a hexmap must be developed in which each patch

hexagon is assigned a nonzero value (typically a 1), and every non-patch hexagon is set to 0. The second step involves assigning each patch a unique ID.

Let's begin with the creation of an initial patch map. This can be done by (1) making a copy of Habitat.csv, which we developed earlier, (2) opening this copy in a spreadsheet application, (3) deleting all of the rows that correspond to hexagons having scores less than 9, and (4) saving the result. Note that I've suggested hexagons scored 9 (the maximum in Habitat.csv) should make up the patches, but that assertion is arbitrary; users are free to create patches any way they see fit. Here, let's assume that this initial patch map has been assigned the filename "Patch_Data.csv".

Next, we will convert Patch_Data.csv into a true patch map by assigning each patch a unique integer ID. To do this, every hexagon making up a patch must be set equal to the patch's ID. This task can be accomplished using the utility *assign_patch_id*. I named the resulting hexmap Patches.csv. A reproduction of the commands I issued to construct Patches.csv is displayed below. Before actually issuing the command, I first make the *assign_patch_id* utility display its common-line arguments.

```
CMD-07:    >> asign_patch_id

              Usage: assign_patch_id  input_file  output_file  binary
                                      random   rows  min_cols  max_cols

CMD-08:    >> assign_patch_id Patch_Data.csv Patches.csv 1 0 2886 2499 2499

              Processing Patch:      168
```

As can be seen above, the *assign_patch_id* utility must be provided with the following parameters:

- Input filename
- Output filename
- Binary-flag
- Randomization-flag
- Rows, min_cols, max_cols

The binary-flag and randomization-flag values must be either 0 or 1. Providing a binary-flag value of 1 instructs the utility to begin by converting all nonzero input map values to 1's. This is useful if the initial patch map contains floating

point numbers and/or negative values; but, it also does no harm when used with a simple input map like ours.

A randomization-flag value of 1 instructs the utility to randomize the patch IDs, which can be useful when a map has lots of patches. When converted to an image, patch colors will be assigned based on IDs, and patch IDs will initially be sequential. Thus, neighboring patches can end up being displayed using similar or even identical colors. Randomization adds visual contrast, making it easier to resolve nearby patches. I have provided a utility called *randomize_patches* for this purpose. The advantage of keeping the patch IDs sequential is that it makes locating specific patches easier, since they are all displayed in order. We won't randomize our patch map, but we will use *randomize_patches* later on, after we create an image of emergent connectivity clusters.

Finally, the *assign_patch_id* utility must be supplied with the hexmap geometry. Once constructed, the Patches.csv hexmap can be visualized using *csv2sun* and *XnView*, as discussed above (Fig. S2, left).

Patches.csv was extracted from Habitat.csv. I mentioned earlier that a patch map need not have any relationship to the other spatial data being used. To reinforce this point, I constructed an alternative patch map that could also be supplied to LINK (Fig. S2, right). To be valid, this new patch map (an array of 25 square patches) must have exactly the same geometry as the hexmap used to simulate movement, which in our case is going to be Habitat.csv (see below). We'll see below that the *run_movement* algorithm will initially place movers into the highest scoring hexagons in Habitat.csv -- exactly the hexagons that comprise Patches.csv. If we use the alternative patch map to develop a competing connectivity analysis, then to the extent that our patch maps don't overlap, individual movers' initial locations will no longer correspond to patch hexagons. But this won't matter, since LINK ignores any portion of a movement path that precedes an individual's arrival at its first patch hexagon. Using an alternative patch map (e.g., Fig. S2, right) will alter the results of a LINK connectivity analysis, but the new results will still be valid and meaningful.

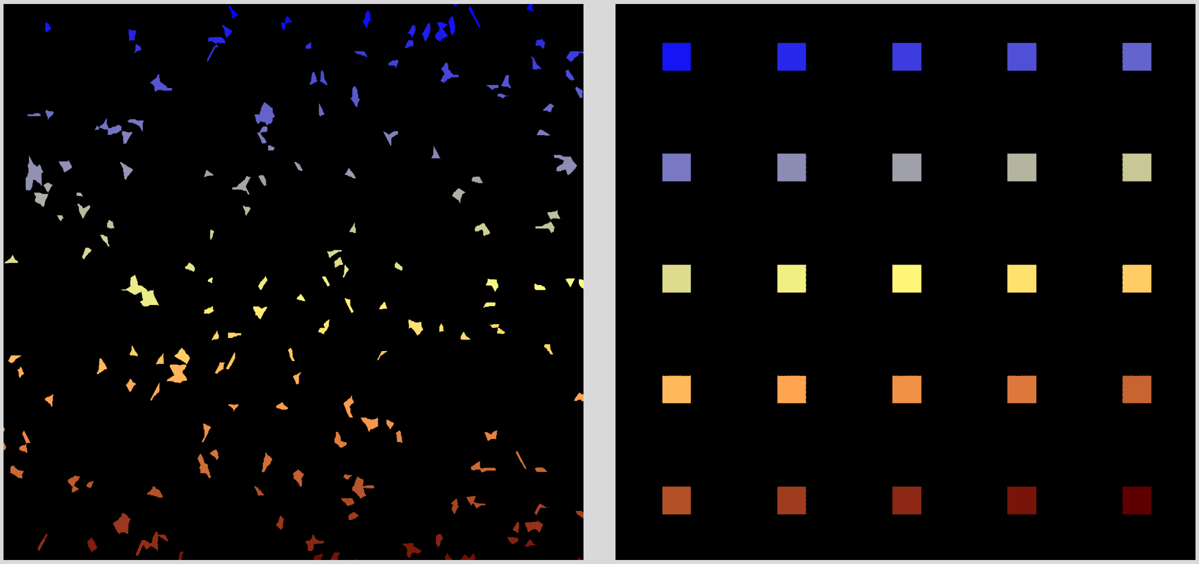

Figure S2. An image of Patches.csv (left) and of an alternative patch map (right). The alternative patch map will not be used in this discussion.

Step C5. Simulating Movement

The next step in the sequence involves simulating individuals moving about in the Habitat.csv hexmap that we built in Step C2. This movement simulation process is required because LINK's connectivity assessment methodology is based upon the analysis of movement data. Movement simulations may be conducted using *HexSim*, as I did for the Fender's blue butterfly case study. Besides being powerful and flexible, *HexSim* has the added advantage that it includes a robust system for simulating movement barriers. Here, we'll instead employ my *run_movement* utility which is not sophisticated at all. Readers should think of *run_movement* as a template rather than a meaningful solution.

LINK imposes two constraints imposed upon movement simulators:

- All movements must involve a series of steps from a hexagon to one of its immediate neighbors (individuals must not jump).
- The output movement records must be properly formatted.

The format required by LINK for movement information is that utilized by *HexSim*. When *HexSim* runs, it generates output called "log files". Log files

record information about every aspect of a simulation. One log file record type is called a “dispersal record”, and is denoted using the keyword “DSP”.

When LINK reads a *HexSim* log file, it ignores all of the file’s content except for the DSP records. Thus, LINK can easily read files created outside of *HexSim*, as long as they contain properly formatted dispersal records. Suitable DSP records must have the following structure:

```
DSP , Replicate , Time Step , Population ID , Individual ID,
      Trait Index , Hexagons Dispersed , Meters Displaced ,
      Hexagons in Path , Path Hexagons
```

Above, spaces have been added for visual clarity. Actual movement records do need to be comma-separated, and should not include spaces. Each of these fields is described briefly in Table S2.

The *run_movement* utility will write out DSP records in the above format. The utility must be passed the following information on the command line:

- The name of a hexmap within which movement will be simulated.
- A name to be assigned to the output CSV file holding the DSP data.
- The number of movers to simulate.
- Each mover’s path length, in hexagons.
- Rows, min_cols, max_cols.

To generate movement data for this example, I ran the following command:

```
CMD-09:    >> run_movement  Habitat.csv  Move_Data.csv
                            5000000 100 2886 2499 2499
```

This instructed the utility to read the Habitat.csv hexmap, and to create an output file named “Move_Data.csv". The *run_movement* utility identified the highest valued hexagons in Habitat.csv, randomly placed five million individuals into those sites, and instructed each of these individuals to move a total of 100 steps. As discussed above, the Habitat.csv hexmap is integer-valued, has a maximum hexagon value of 9, and is arranged into 2886 rows and 2499 columns (in both odd and even rows). Readers who’d prefer that *run_movement* proceed more rapidly may ask it to place fewer individuals into the landscape.

Table S2. A description of *HexSim*'s DSP data records, which are read by LINK.

ITEM	TYPE	USAGE	INTERPRETATION
DSP	String	Keyword	Identifies dispersal records.
Replicate	Integer	Used	Distinguishes data generated during multiple replicate simulations.
Time Step	Integer	Used	Used to join movement records belonging to the same individual.
Population ID	Integer	Ignored	LINK assumes all DSP records belong to a single population.
Individual ID	Integer	Used	Identifies individual DSP records. IDs need only be unique within a replicate.
Trait Index	Integer	Ignored	Specific to *HexSim*.
Hexagons Dispersed	Integer	Ignored	Specific to *HexSim*.
Meters Displaced	Floating Point	Ignored	Specific to *HexSim*.
Hexagons in Path	Integer	Used	Indicates the number of hexagons that will be listed in the data that follows.
Path Hexagons	List of Integers	Used	Lists the IDs of visited hexagons. The first ID must be the movement starting point.

As mentioned above, the *run_movement* algorithm is very simple. Each individual begins by selecting a preferred direction for its movement. A maximum of six preferred directions are available, corresponding to the hexagons immediately adjacent to an individual's current location. Later, individuals are allowed to change their preferred direction when zero-valued hexagons or map edges are encountered. As they move, individuals alternate between selecting their next hexagon based upon their preferred direction, or instead choosing the highest quality neighboring site. That's all it does.

D. Using LINK to Evaluate Connectivity

In this section, we'll walk through a sequence of six connectivity assessment tasks performed using the LINK utility. Accordingly, LINK has six individual functional modes, which users select by specifying a "function code" on the command line (see Table S3). Function codes are integers between 1 and 6. LINK will write out a usage statement if it is called with no arguments. LINK will also display six additional usage statements when called with a valid function code, but insufficient other arguments. I do not display these usage statements below.

Step D1. Mapping Potential Connectivity

In Step C5, we generated a file containing precisely-formatted movement records. Here, we use LINK to convert this data into a hexmap that records potential connectivity across the landscape. Then, we'll construct an image from the hexmap. The first step is performed by calling LINK using the following command-line data:

- A function code of 1.
- The name of an input file containing movement data.
- A name to be assigned to the output hexmap.
- The geometry of the hexmap used with the movement simulator.

The function code of 1 instructs LINK to build a potential connectivity hexmap from DSP data records. We'll use "Move_Data.csv" as the input filename (see Step C5). I selected "Potential_Connectivity" as an output filename. Note that LINK expects input filename extensions to be provided, but it adds output extensions automatically. This frees users from needing to remember which extension is correct for each of LINK's multiple output files. The Habitat.csv hexmap, which we used to run the movement simulator, has 2886 rows and 2499 columns (in odd and even rows). Thus, the actual command we want to issue is:

```
CMD-10:    >> LINK 1 Move_Data.csv Potential_Connectivity 2886 2499 2499
```

It may take some time to complete this command; processing speed will depend on the number of movement records contained in the input file. Next, we want to visualize the Potential_Connectivity.csv result that was just generated. The

utilities I use to convert CSV files into images impose a linear relationship between hexagon score and color. If a hexmap has a few very high values (which is common when recording movement), then most hexagons will be assigned colors from the "low" end of the colormap, and the resulting image will have little contrast. My utility called *bin_hexmap* solves this problem by sorting hexagons by score, and then placing them into equally populated bins.

Table S3. The LINK utility's six function codes, and their meanings.

CODE	DATA IN	DATA OUT	DESCRIPTION
1	Movement Data	Potential Connectivity	Uses properly formatted movement data to create a hexmap illustrating potential connectivity. The input may be a HexSim log file, or a file that contains DSP records.
2	Movement Data	DSP Data Table	Combines movement records in order to create a compact LINK input data file.
3	DSP Data Table	Linkages Table	Extracts just the portions of movement records that link resource patches.
4	Linkages Table	Connectivity Reports	Builds a dispersal kernel, a connectivity cluster report, and hexmaps showing connected and isolated resource patches.
5	Linkages Table	Linkages Table	Extracts a subset of a Linkage Table that only contains data for movements from resource patch X to resource patch Y.
6	Linkages Table	Realized Connectivity	Creates a hexmap visualizing realized connectivity, or a hexmap displaying any emergent connectivity clusters.

The *bin_hexmap* utility must be passed the name of an input CSV hexmap, and the number of bins to use. All of my imaging utilities assume 8-bit colormaps, so the number of bins must be less than 256. I typically supply a value of 250, leaving 5 colormap slots for later use. The *bin_hexmap* utility writes to the standard output, and it should be used with unix-style redirection. I redirected

the output into a file named “Potential_Connectivity_Binned.csv”, using following command (which was actually all placed on a single line):

```
CMD-11:    >> bin_hexmap Potential_Connectivity.csv 250 >
                         Potential_Connectivity_Binned.csv
```

Finally, either *csv2sun.c* or *csv2png.py* can be used to generate an image from the CSV hexmap. The use of these utilities has been discussed above. I used csv2sun to build a Sun Raster file, and then used *XnView* to create a PNG file named “Potential_Connectivity_Binned.png”. Fig. S3 helps to illustrate the value of the *bin_hexmap* utility.

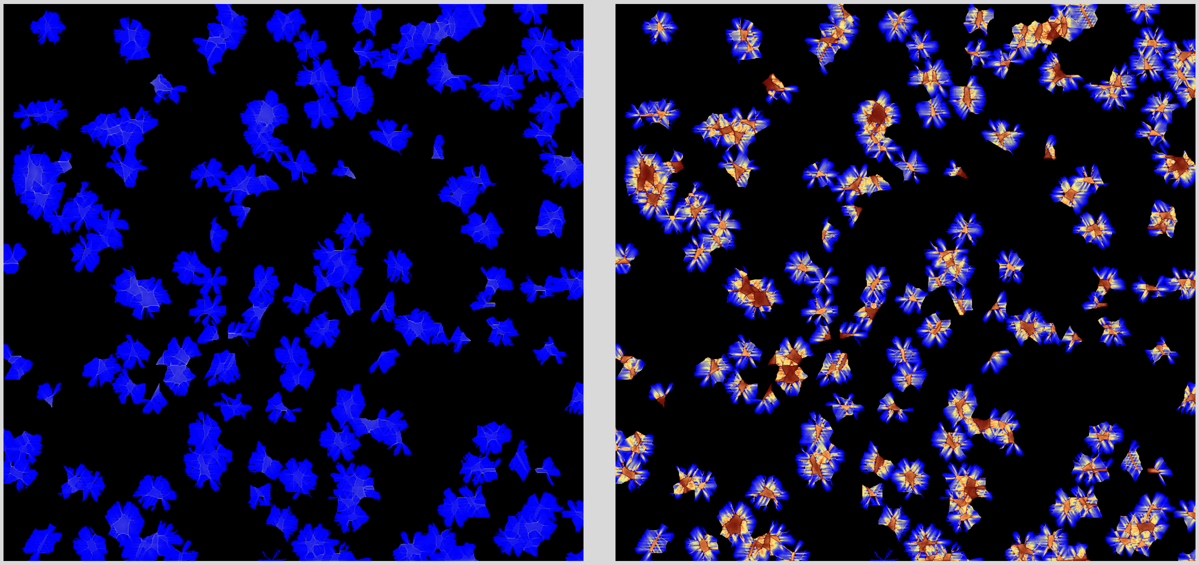

Figure S3. Image of LINK’s emergent potential connectivity hexmap before (left) and after (right) enhancement via the *bin_hexmap* utility.

Step D2. Building a DSP Data Table

A DSP data table is a condensed and reformatted version of a *HexSim* log file, or more generally, of any collection of DSP records. LINK constructs DSP data tables by locating and joining all of the distinct movement records attributable to a single individual. See Step C5 for the required DSP record format. Once LINK has converted a file of movement data into a DSP data table, the original file containing DSP data is no longer needed.

Using LINK to construct a DSP Data Table requires supplying the following command-line data:

- A function code of 2.
- An input file containing properly-formatted movement data.
- A name to be assigned to the output DSP Data Table.

- The number of distinct individuals represented in the movement data.
- The number of simulation time steps contained in the movement data.
- The maximum number of separate movements made per time step.
- The maximum distance (in hexagons) traveled in a movement event.

The first three of these command-line inputs are always required. The last four input values must only be supplied when the input file contains multiple DSP movement records per individual. In such cases, all of the distinct records associated with a single individual must be identified and merged into one continuous record. The final four parameters simplify this merging process.

The *run_movement* utility only moved individuals a single time. Thus, I was free to issue the simpler version of the command that creates a DSP Data Table:

```
CMD-12:    >> LINK 2 Move_Data.csv DSP
```

In the above command, the "2" instructs the LINK utility to build a DSP Data Table. Move_Data.csv is the name of the file that was produced by *run_movement* in Step C5. And "DSP" is an arbitrary name I picked for the DSP Data Table. LINK will add a ".txt" extension to the resulting output file.

DSP Data Tables have a very simple format. They are just a collection of lines, with each line containing the following information:

N_i H_1 H_2 H_3... H_N

Each line represents the entire movement path for a single individual. N_i (above) represents the number of hexagons listed on line N, and H_1 , H_2, ..., H_N are hexagon IDs. Developing a DSP Data Table is trivial if each individual only moves once, but the process becomes more involved when individuals move multiple times, movement distances are variable, or simulations are replicated.

Step D3. Constructing a Linkage Table

Linkage Tables are constructed from DSP Data Tables, and they only contain movement data for the portions of paths that connect pairs of focal patches. Users can build alternative Linkage Tables from the same DSP Data Table by supplying multiple patch maps; every distinct patch map will produce a unique Linkage Table. LINK's ability to utilize multiple patch maps will help users examine how sensitive their conclusions about landscape connectivity are to assumptions that govern the size, shape, and distribution of resource patches.

Using LINK to construct a Linkage Table requires supplying the following command-line data:

- A function code of 3.
- A DSP Data Table.
- A name to be assigned to the output Linkage Table.
- The name of a patch map.
- The patch map's geometry.

The patch map's geometry must be the same as that of the spatial data used to simulate movement; but otherwise these files need not be related.

I used the following command to construct a Linkage Table from the data assembled above:

```
CMD-13:    >> LINK 3 DSP.txt LNK Patches.csv 2886 2499 2499
```

In the above command, the "3" instructs the LINK utility to build a Linkage Table. DSP.txt is the name of the DSP Data Table generated previously. And "LNK" is an arbitrary name I selected for the Linkage Table. A ".txt" extension will be added automatically to the resulting output file. Patches.csv is the name of the patch map we constructed in Step C4, and the remaining parameters specify the map geometry. Recall that each patch must be assigned a unique patch ID. A common error at this step is to supply LINK with a binary patch map rather than a true patch map.

Linkage Tables have two distinct record types, though they really all have the same underlying structure. When an individual has successfully moved between two patches, a record will be generated that has the following format:

X_i, Y_i, N_i H_1 H_2 H_3… H_N

Here, X_i and Y_i represent the starting and ending patch IDs associated with movement path i. N_i represents the number of hexagons in path i, and H_1 , H_2, …, H_N represent hexagon IDs.

A different record formatting structure is used when:

- A movement begins and ends in the same patch.
- A movement begins in a patch, but ends outside of any patches.

For both of these record types, the effective path length will be zero (because these paths don't connect distinct patches). But it is still important to record these events so that LINK has the information it needs to construct a dispersal kernel. In these cases, a record will be generated that appears as follows:

X_i, Y_i, 0

Here, X_i and Y_i represent the starting and ending patch IDs associated with movement path i. However, Y_i must always equal either X_i or zero. X_i and Y_i will be the same when an individual begins and ends in the same patch, and Y_i will be zero when an individual stops moving in a non-patch hexagon. When Linkage Tables are constructed, LINK discards any movement data prior to an individual's arrival at its first patch. Linkage Tables therefore never include records for which X_i is zero.

Step D4. Assembling the Connectivity Reports

Once a Linkage Table has been developed, the next step is to generate LINK's connectivity reports. To do so, LINK must be passed the following parameters:

- A function code of 4.
- A Linkage Table.
- A prefix to be assigned to the output files.
- A patch map.
- The patch map's geometry.

I used the following command to generate LINK's four connectivity reports:

```
CMD-14:    >> LINK 4 LNK.txt Result Patches.csv 2886 2499 2499
```

In the above command, the "4" instructs the LINK utility to build connectivity reports. LNK.txt is our Linkage Table, and I elected to use the prefix "Results" for the output files. Patches.csv is the name of our patch map, and the remaining values specify that map's geometry.

When invoked this way, LINK will create four separate connectivity reports, each of which will be formatted as a CSV file:

- A list of all connectivity clusters, with suffix : *-clusters*
- An emergent dispersal kernel, with suffix : *-matrix*
- A hexmap of isolated patches, with suffix : *-patches [isolated]*
- A hexmap of connected patches, with suffix : *-patches [connected]*

The table of connectivity clusters identifies which patches are functionally connected to each other, and how much movement traffic was associated with each cluster. The dispersal kernel stores the probabilities that individuals will move from a patch to any other patch (including itself). The hexmaps displaying isolated and connected patches can be useful when developing visualizations of model outcomes.

Importantly, when LINK is used to develop maps of Connectivity Clusters (see Step D6, below) users will have the option to use a map of connected patches (the final item in the above bullet list) in place of the standard patch map.

My report titled "Result-clusters.csv" recorded a total of 32 connectivity clusters. The number of patches per cluster ranged from 2 to 8. Cluster traffic varied between 2 and 21,362 realized connectivity events.

The emergent dispersal kernel, titled "Result-matrix.csv" is sparse (as expected), and contains 168 rows and columns, corresponding to the 168 patches in our patch map. As discussed above, I used *csv2sun* and *XnView* to convert the two patch maps into PNG image files (Fig. S4).

Step D5. Extracting Connectivity Data

When generated as described in Step D4, Linkage Tables will store the portions of every movement path that connect any possible pair of patches. Users will sometimes find it useful to extract subsets of this linkage data corresponding to movements between specific patches. This could be done using a spreadsheet, but the LINK utility provides a simple way for readers to accomplish this task. Specifically, the LINK utility can extract specific records from a Linkage Table, and write the results to a new, smaller Linkage Table. To do so, LINK must be passed the following parameters:

- A function code of 5.
- The name of an input Linkage Table.
- A name for the output Linkage Table.
- The starting patch ID.
- The ending patch ID.

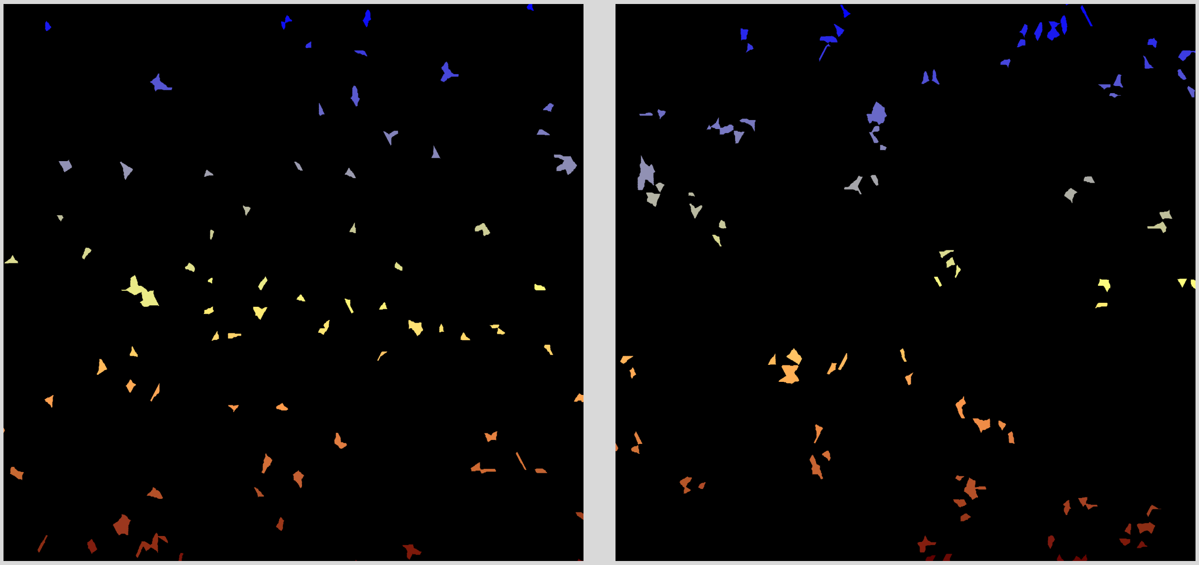

Figure S4. Images of the hexmaps corresponding to the CSV files named "Results-patches [isolated]" (left) and "Results-patches [connected]" (right).

I used the following two commands to isolate the movement paths beginning at patch 39 and ending at patch 40, and visa-versa:

```
CMD-15:    >> LINK 5 LNK.txt Linkages_39_to_40 39 40
CMD-16:    >> LINK 5 LNK.txt Linkages_40_to_39 40 39
```

In the above commands, the "5" instructs the LINK utility to extract a subset of a Linkage Table. Obviously, "Linkages_39_to_40" and "Linkages_40_to_39" are the names I selected for the two output files. Finally, each command ends with the intended starting and ending patch IDs. The new output files are valid Linkage Tables, but they are much smaller than LNK.txt.

Running these commands makes LINK extract all of the input linkage data corresponding to paths that begin at the starting patch and end at the ending patch. The output Linkage Tables can then be used to produce connectivity maps that illustrate these specific emergent movement pathways. The process of constructing hexmaps from Linkage Tables is discussed in Step D6, below.

Step D6. Visualizing Realized Connectivity

Finally, LINK is able to map both realized connectivity and connectivity clusters. Realized connectivity data are stored in Linkage Tables, which LINK can convert to hexmaps. LINK can also use Linkage Tables to map connectivity clusters; these maps are visual analogs of the connectivity cluster reports described in Step D4. When using LINK to image connectivity clusters, users will need access to a hexmap that records connected patches (see Fig. S4).

To invoke these LINK algorithms, a function code of 6 must be supplied. When this is done with no other parameters being supplied, LINK will return the following usage statement:

```
CMD-17:    >> LINK 6
              Usage:  LINK 6  linkage_table  output_file  goal  [patch map]
                      rows  min_cols  max_cols
```

When building a realized connectivity hexmap from a Linkage Table, it is necessary to use a goal of 0. In such cases, a patch map will not be used.

When building a hexmap of connectivity clusters, the goal must be set to 1, and a patch map has to be specified. For the best results, this patch map should

contain only connected patches (otherwise, hexmap visualizations of the connectivity clusters will include isolated patches). We built such a patch map in Step D4, above. Note that the patch map must be in CSV format.

To summarize, when constructing a map of realized connectivity, LINK must be passed the following command-line parameters:

- A function code of 6.
- The name of the input Linkage Table.
- A name for the output hexmap.
- An algorithmic goal of 0.
- The Linkage Table geometry.

When constructing a map of connectivity clusters, LINK must be passed the following command-line parameters:

- A function code of 6.
- The name of the input Linkage Table.
- A name for the output hexmap.
- An algorithmic goal of 1.
- A patch map, preferably containing only connected patches.
- The Linkage Table geometry.

For this illustration, I instructed LINK to construct a realized connectivity hexmap using the following command:

```
CMD-18:    >> LINK 6 LNK.txt Realized_Connectivity 0 2886 2499 2499
```

I then used the *bin_hexmap* utility to develop a modified version of Realized_Connectivity.csv, which I named Realized_Connectivity_Binned.csv. Then, I used *csv2sun* and *XnView* to develop a PNG image of the data stored in Realized_Connectivity_Binned.png (Fig. S5, left).

Next, I asked LINK to construct a hexmap illustrating the emergent connectivity clusters. To accomplish this, I used the following command:

```
CMD-19:    >> LINK 6 LNK.txt Connectivity_Clusters 1
                     Results-patches\ \[connected\].csv 2886 2499 2499
```

The “\” characters in the above patch map name prevent *Cygwin* from interpreting the space and brackets as special characters, which would cause them to be assigned unintended meanings.

LINK builds maps of connectivity clusters by superimposing the movement paths contained in the Linkage Table on top of the patch map passed on the command line. This explains why this patch map shouldn’t include isolated patches -- the isolated patches will not match up to any of the connected movement paths in the output image.

Having done this, I randomized the cluster IDs using my *randomize_patches* utility. The syntax I used for *randomize_patches* is as follows:

```
CMD-20:    >> randomize_patches Connectivity_Clusters.csv >
                                Connectivity_Clusters_Random.csv
```

Note that the above command was entered on a single line in *Cygwin*. Finally, I generated a PNG image of Connectivity_Clusters.csv (Fig. S5, right).

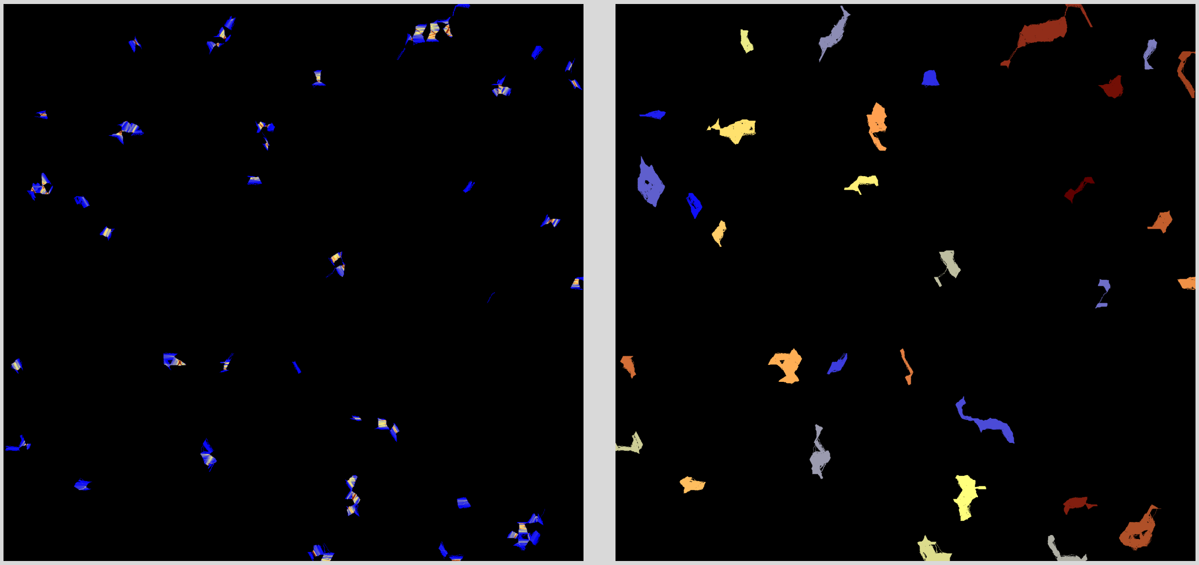

Figure S5. Realized connectivity (left) and connectivity clusters (right). The connectivity clusters map is a combination of realized connectivity and the patch map supplied to LINK. Here, the connectivity cluster IDs have been randomized to improve contrast.

Recall that, in Step D5, we used LINK with a function code of 5 to develop Linkage Tables containing only the movement paths that connected patches 39 and 40. I subsequently used LINK with a function code of 6 (as shown above) to create hexmaps that separately illustrate the movements from patch 39 to 40, and from patch 40 to 39. Then, I used the *bin_hexmap* and *csv2sun* utilities, as well as *XnView* to convert these CSV hexmaps into PNG images. These specialized connectivity maps are shown in Fig. S6, juxtaposed with an image of realized connectivity for the entire cluster formed by patches 39, 40, and 43.

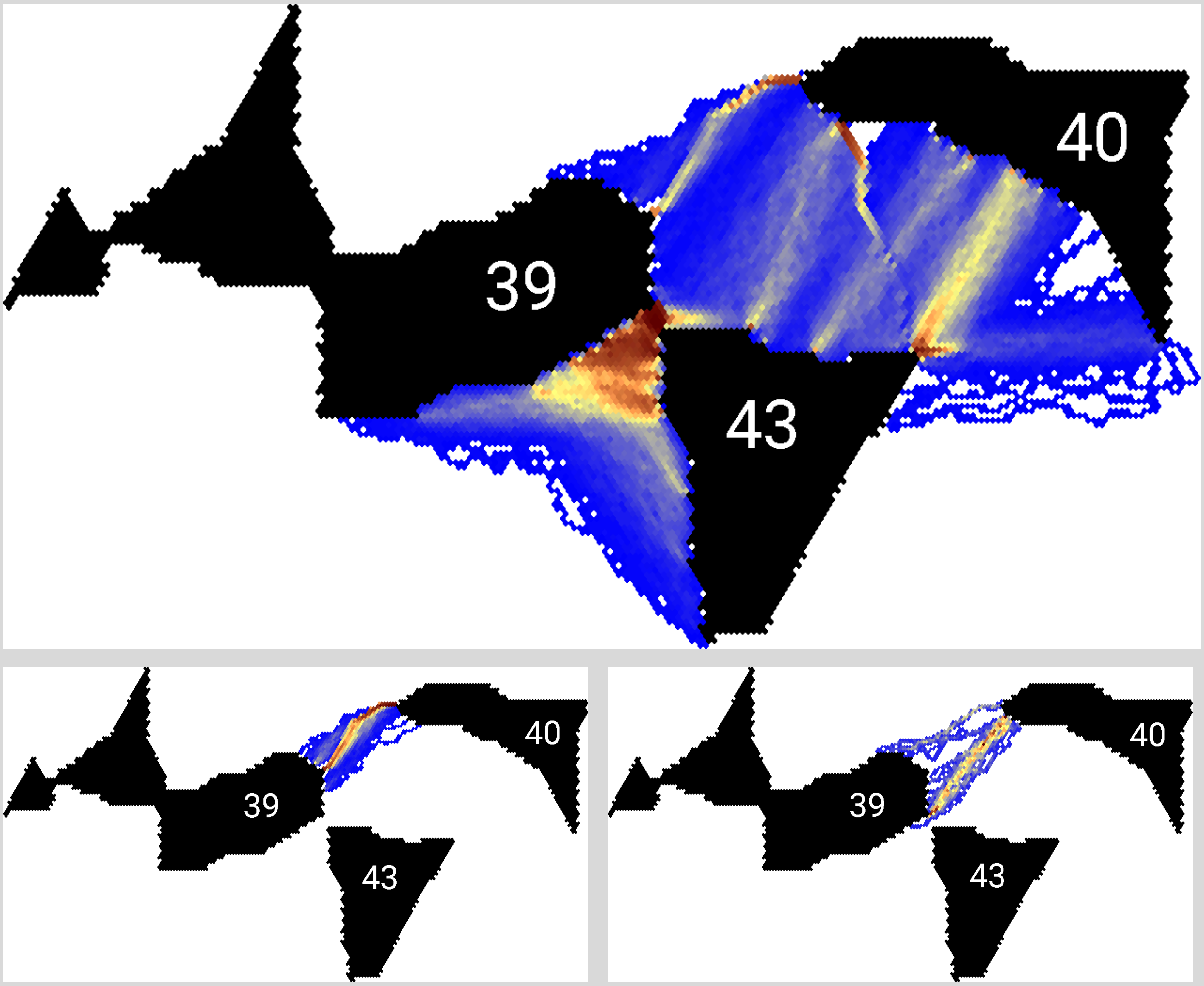

Figure S6. Traffic within the connectivity cluster formed by patches 39, 40, and 43. White labels indicate the patch IDs. Top: All of the movement paths connecting the cluster's patches. Bottom-left: Movements from patch 39 to patch 40. Bottom-right: Paths leaving patch 40 and reaching patch 39.

Fig. S6 suggests that, even in a simple system like this, patch-specific contributions to realized connectivity can be notably asymmetric. The majority of the traffic in this connectivity cluster is clearly to, or from, patch 43. The cluster traffic from patch 39 to patch 40 also appears higher than that in the opposite direction.

E. Concluding Thoughts

This document is intended to serve as a guide to LINK and its companion utilities. This discussion made use of a fabricated land cover map (Habitat.asc) because it is simple but not trivial, and small enough to be easy to work with.

Above, we have walked through an example of the entire connectivity assessment process, beginning with a land cover map, and ending with (1) maps of potential and realized connectivity, (2) a connectivity cluster analysis, (3) a map of the connectivity clusters, (4) an emergent dispersal kernel, and (5) maps indicating which resource patches are connected and which are isolated. We have also examined how LINK can be used to extract and visualize movement paths that connect specific pairs of resource patches.

Landscape data is almost always represented using arrays of square pixels. Thus, it is critical that LINK's connectivity assessment workflow begins and ends with raster imagery. It does. Early in our analysis, we tessellate the input raster maps using a grid of hexagonal cells. This allows movement to be simulated without introducing artifacts -- unlike in square grids, each of a hexagon's neighbors are equidistant. Eliminating these artifacts disambiguates inferences drawn from LINK's measures of functional landscape connectivity. Regardless, the outputs from this workflow are tables and raster images. Readers working with raster maps should find LINK's connectivity analysis straightforward, and its results compatible with their existing imagery.

To make the analysis here possible, I have provided readers with a suite of software utilities. These are carefully tested and well-used algorithms, but they are somewhat unsophisticated and at times redundant, were developed using the relatively unfriendly C programming language, and could benefit from modernization and extension (in multiple ways). I encourage interested readers to improve upon these tools, and to make any updates available to others.
